# Isovalerylspiramycin I Reprograms the Immunosuppressive and Temozolomide-Resistant Microenvironment by Inhibiting the Frizzled-5/Wnt/β-Catenin Pathway in Glioblastoma

**DOI:** 10.34133/research.0828

**Published:** 2025-08-13

**Authors:** Xin Luo, Xiangyang Zhong, Tianci Zeng, Xiaodie Li, Tao Yang, Qu Yue, Yufei Lan, Sui Chen, Zhao Wang, Manqing Zhang, Boming Zuo, Yuankai Wang, Yixiong Shen, Jiankun Lu, Boyang Liu, Hongbo Guo

**Affiliations:** ^1^ Department of Neurosurgery, The National Key Clinical Specialty, The Engineering Technology Research Center of Education Ministry of China on Diagnosis and Treatment of Cerebrovascular Disease, Guangdong Provincial Key Laboratory on Brain Function Repair and Regeneration, The Neurosurgery Institute of Guangdong Province, Zhujiang Hospital, Southern Medical University, Guangzhou 510282, China.; ^2^ Department of Neurosurgery, Department of Neuro-oncological Surgery, The National Key Clinical Specialty, The Engineering Technology Research Center of Education Ministry of China on Diagnosis and Treatment of Cerebrovascular Disease, Guangdong Provincial Key Laboratory on Brain Function Repair and Regeneration, The Neurosurgery Institute of Guangdong Province, Zhujiang Hospital, Southern Medical University, Guangzhou 510282, China.

## Abstract

Glioblastoma (GBM), the most prevalent and lethal primary brain malignancy in adults, currently lacks treatment effective options. Repurposing existing pharmaceutical agents as novel therapeutic modalities represents a viable strategy for efficiently utilizing resources. Here, we demonstrated that Isovalerylspiramycin I (ISP-I), the active component of a novel macrolide antibiotic, exerts a synergistic effect with temozolomide (TMZ) to enhance anti-GBM efficacy. ISP-I potently induced cytotoxicity and apoptosis through the induction of DNA double-strand breaks. The synergistic activity (combination index < 1) was confirmed for ISP-I in combination with TMZ against GBM. Additionally, ISP-I was found to induce immunogenic cell death, as evidenced by increased adenosine triphosphate release and calreticulin exposure. In murine models, ISP-I increased tumor-infiltrating CD8^+^ T cells, enhanced effector subsets, and reduced exhausted subsets. Mechanistically, ISP-I targeted the Frizzled-5 (FZD5)/Wnt/β-catenin signaling pathway, resulting in suppression of GSK-3β phosphorylation. This event subsequently increased β-catenin phosphorylation, reducing its translocation into the nucleus. Consequently, the binding of transcription factors (T-cell factor 1/lymphoid enhancer factor 1) to promoters of *CD274* and O^6^-methylguanine-DNA methyltransferase (*MGMT*) was impeded, thereby enhancing GBM cell susceptibility to TMZ. These findings elucidate the mechanisms underlying ISP-I’s therapeutic efficacy in GBM and provide essential evidence for its clinical translation and combinatorial therapeutic strategies.

## Introduction

Glioblastoma (GBM) represents the most prevalent, highly invasive primary intracranial malignancy, characterized by a profoundly elevated mortality rate [1]. The first-line chemotherapeutic agent, temozolomide (TMZ), kills tumor cells by generating cytotoxic DNA lesions resulting from O^6^-guanine alkylation. However, cells expressing O^6^-methylguanine-DNA methyltransferase (MGMT) exhibit substantial resistance to TMZ, which represents a critical cause of therapeutic failure [2,3]. Consequently, the development of drugs that can restore TMZ sensitivity relies heavily on targeting MGMT [4–6]. The antigen-specific CD8^+^ T-cell activation, proliferation, and effector function are inhibited through programmed cell death protein 1 (PD-1) engagement. Such interactions allow cancer cells to evade immune destruction [7–9]. In GBM tissues, immune checkpoint molecules exhibit marked overexpression, which correlates with poor clinical prognosis [10,11]. The programmed cell death ligand 1 (PD-L1) expression is markedly up-regulated in relapsed GBM patients [12–14] and further increased in GBM lesions following administration of TMZ and radiotherapy [15,16]. Characterizing PD-L1 as a potential biomarker holds promise for predicting treatment response and prognosis in GBM. Reducing PD-L1 expression may reduce immune evasion and improve glioma patient outcomes [17,18]. Consequently, a promising therapeutic strategy for the treatment of GBM is simultaneously targeting MGMT and PD-L1.

The Wnt/β-catenin signaling axis mediates both therapeutic resistance and immunosuppressive adaptations in GBM. Cytoplasmic accumulation and nuclear translocation of β-catenin define pathway activation, driving transcription of downstream oncogenic genes [19–22]. This pathway is associated with immune exclusion phenotypes in multiple cancers [23,24] and is aberrantly activated following TMZ treatment, contributing to chemoresistance [20,25–27]. Pathway activity correlates with the blood–brain barrier (BBB) integrity, and its inhibition enhances TMZ delivery and tumor sensitivity [28]. Crucially, this pathway governs the transcriptional regulation of *MGMT* and *CD274* (PD-L1) genes, underscoring its dual role in mediating drug resistance and immune evasion [26,29–32].

To address the challenges in GBM treatment, novel drugs are being explored. Notable among these candidates is Carrimycin, a novel macrolide antibiotic recently synthesized and granted approval for clinical use [33,34]. Its active component, Isovalerylspiramycin I (ISP-I), inhibits growth of hepatocellular carcinoma and oral squamous cell carcinoma. ISP-I has been demonstrated to target selenoprotein H in the nucleus, suppressing the growth of tumors and metastases [33,35,36]. However, the impact of ISPI on TMZ sensitivity and its potential for immune modulation in GBM remains unclear.

This study demonstrated that ISP-I suppresses tumor cell proliferation while eliciting immunogenic cell death (ICD) and DNA damage in GBM. Furthermore, the combination of ISP-I and TMZ demonstrated synergistic inhibitory effects on GBM growth. Additionally, ISP-I was shown to augment the antitumor capabilities of effector T lymphocytes in GBM models. Mechanistically, ISP-I exerted inhibition of Frizzled-5 (FZD5)-mediated Wnt/β-catenin signaling, effectively impeding the β-catenin nuclear translocation, and disrupting TCF1/LEF1 binding to the promoters of *CD274* and *MGMT*. These findings suggest that ISP-I may possess anti-GBM properties and the potential to overcome resistance to TMZ and immunosuppressive issues in GBM patients. This provides essential evidence for the clinical application of combination therapy strategies.

## Results

### ISP-I induces cytotoxicity via DNA damage in GBM cells and enhances TMZ sensitivity in vitro

**Fig. 1.**
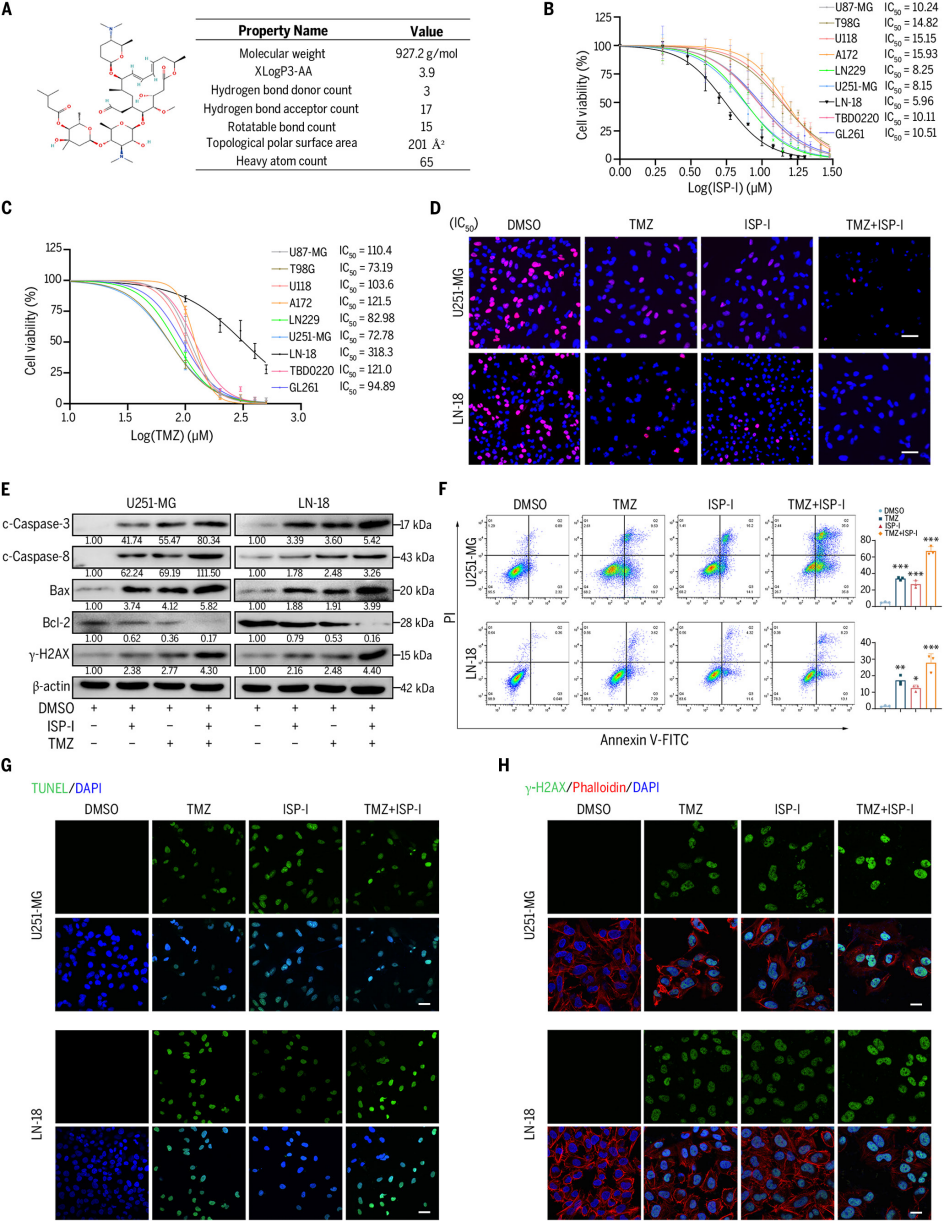
ISP-I induces cytotoxicity via DNA damage in GBM cells and enhances TMZ sensitivity in vitro. (A) Chemical structure of ISP-I. (B and C) GBM cell lines were treated with ISP-I (B) or TMZ (C) for 24 h, and the IC_50_ values were calculated by the CCK-8 assay (*n* = 3). (D) The EdU proliferation assays were used on GBM cells after 24 h cotreatment with ISP-I and TMZ at their respective IC_50_ concentrations (U251-MG: 8.0 μM ISP-I, 80.0 μM TMZ; LN-18: 5.0 μM ISP-I, 300.0 μM TMZ). Scale bars = 50 μm. (E and F) Apoptosis responses in GBM cells treated with ISP-I and TMZ at IC_50_ for 24 h were analyzed by Western blotting (E) and Annexin V/PI double staining (F, *n* = 3). (G and H) The effect of treatment with ISP-I (IC_50_) and TMZ (IC_50_) on DNA damage was measured by TUNEL assay (G, scale bars = 50 μm) and γ-H2AX immunofluorescence staining (H, scale bars = 20 μm). Data: mean ± SD; significance was calculated with one-way analysis of variance (ANOVA) test. *P < 0.05; **P < 0.01; ***P < 0.001.

The physicochemical properties of ISP-I were depicted in Fig. 1A. Cell Counting Kit-8 assays (CCK-8 assays) were conducted to assess its inhibitory effect on various GBM cell lines (Fig. S1A), including U87-MG, T98G, U118, A172, LN229, U251-MG, LN-18, TBD0220, and GL261. GBM cell proliferation was markedly inhibited by ISP-I after 24-h treatment (Fig. 1B), including U251-MG and LN-18 cells, which exhibited the lowest half-maximal inhibitory concentration (IC_50_ = 8.15 and 5.96 μM, respectively). These two cell lines were selected for subsequent investigations of anti-GBM mechanisms. 5-Ethynyl-2'-deoxyuridine incorporation assays (EdU assays) showed reduced proliferation in ISP-I-treated GBM cells (Fig. S1B). Furthermore, Western blotting demonstrated ISP-I-mediated apoptosis via caspase-3/8 cleavage, Bax up-regulation, and Bcl-2 down-regulation (Fig. S1C), with Annexin V/propidium iodide (PI) staining confirming dose-dependent apoptotic induction (Fig. S1D). Transferase dUTP nick-end labeling (TUNEL) and γ-H2AX immunofluorescence assays were employed to quantitatively assess DNA fragmentation patterns. Mechanistically, TUNEL (Fig. S1E) and γ-H2AX immunofluorescence staining (Fig. S1F) showed that ISP-I induced DNA double-strand breaks.

Given the shared DNA damage-mediated cytotoxicity of ISP-I and TMZ, synergistic effects were evaluated. The IC_50_ values of single-agent ISP-I and TMZ (Fig. 1C) were determined, enabling rational selection of combination ratios of 1:10 for U251-MG (ISP-I:TMZ = 8.0 μM:80.0 μM) and 1:60 for LN-18 cells (ISP-I:TMZ = 5.0 μM:300.0 μM). Synergistic effect quantification via CompuSyn software defined combination index (CI) values < 1 as statistically significant synergy at low concentrations (Fig. S1G). The synergistic antiproliferative effect was detected by EdU assays at respective IC_50_ concentrations of ISP-I and TMZ (Fig. 1D). Western blot analysis for apoptosis markers demonstrated that ISP-I + TMZ treatment markedly induced apoptosis in GBM cells (Fig. 1E). Furthermore, the effect of the TMZ group was superior to that of the ISP-I treatment group. Moreover, Annexin V/PI double staining demonstrated that the combination regimen resulted in a markedly elevated apoptotic rate compared to monotherapy conditions (Fig. 1F). The TUNEL assay (Fig. 1G) and immunofluorescence staining for γ-H2AX demonstrated that the effect of the TMZ treatment group was better than that of the ISP-I group, while the ISP-I + TMZ treatment group enhanced DNA damage compared to the levels observed in the ISP-I alone or TMZ alone groups (Fig. 1H).

### ISP-I down-regulates MGMT expression in vitro and in vivo

**Fig. 2.**
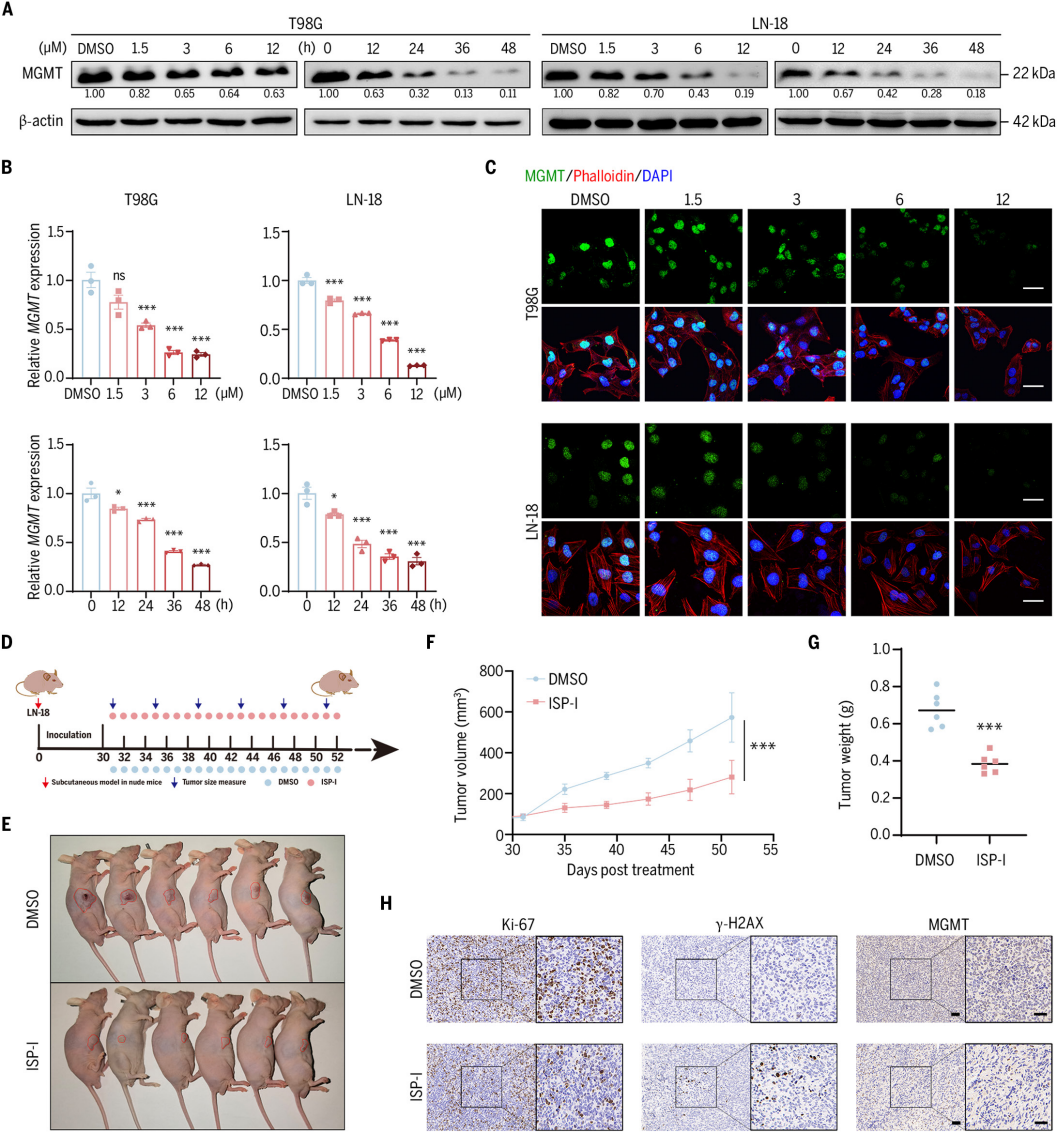
ISP-I down-regulates MGMT expression in vitro and in vivo. (A) The modulatory effect of ISP-I on MGMT protein expression was evaluated by Western blotting in MGMT-positive GBM cells. (B) Quantitative assessment of ISP-I-mediated transcriptional regulation of *MGMT* was performed using qRT-PCR (*n* = 3). (C) The MGMT expression in GBM cells following ISP-I treatment was examined by immunofluorescence staining. Scale bar = 20 μm. (D) Schematic diagram of implantation, growth monitoring, and ISP-I administration. (E) Subcutaneous tumor-bearing mice were treated with DMSO and ISP-I (50 mg/kg) by gavage for 4 weeks, respectively (*n* = 6). (F) Tumor volumes were quantified at 4-day intervals following drug administration. Tumor volume = 0.52 × length × width^2^ (*n* = 6). Significance was calculated with Student’s *t* test. (G) Wet weights of 2 groups of fresh tumors were collected (*n* = 6). Significance was calculated with Student’s *t* test. ***P < 0.001. (H) IHC staining was performed for Ki-67, γ-H2AX, and MGMT in tumor tissues. Scale bars = 20 μm. Data: mean ± SD; significance was calculated with one-way ANOVA test. *P < 0.05; ***P < 0.001; ns, not significant.

In MGMT-positive GBM cells (T98G and LN-18), ISP-I elicited dose- and time-dependent reductions in both MGMT protein levels (Fig. 2A) and mRNA expression (Fig. 2B). Confocal microscopy further revealed a dose-dependent decrease in nuclear MGMT expression with increasing ISP-I concentrations (Fig. 2C). We subsequently constructed a subcutaneous xenograft model in immunodeficient mice using LN-18 cells (Fig. 2D). The results demonstrated a notable reduction in the tumor burden after 4 weeks of ISP-I treatment (Fig. 2E to G). Immunohistochemical analysis revealed that Ki-67 expression and MGMT protein levels were markedly attenuated in the ISP-I-treated group compared with the dimethyl sulfoxide (DMSO) group, while γ-H2AX exhibited an inverse trend (Fig. 2H).

### ISP-I down-regulates PDL1 expression, suppresses orthotopic GBM tumor growth, and synergizes with TMZ

**Fig. 3.**
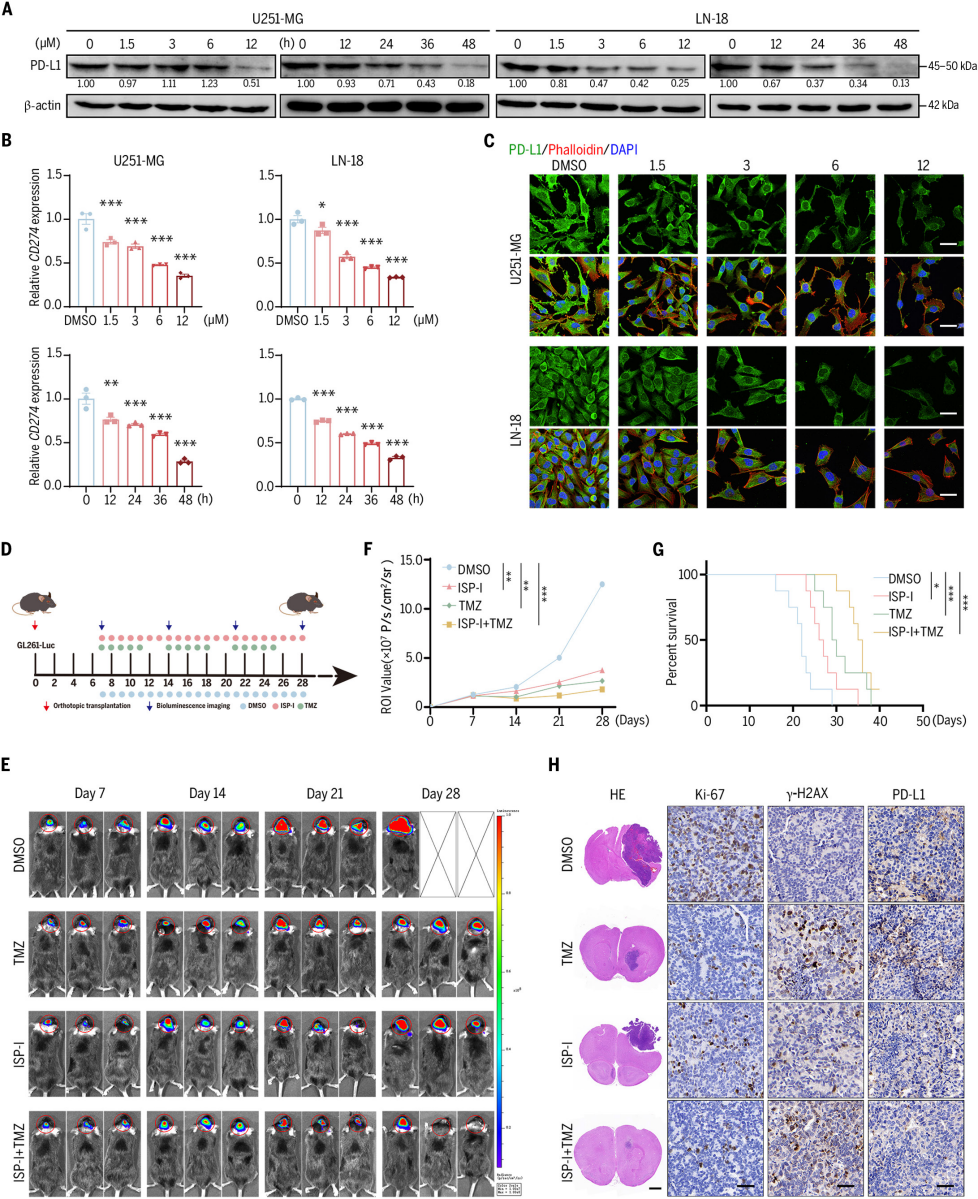
ISP-I down-regulates PD-L1 expression, suppresses orthotopic GBM tumor growth, and synergizes with TMZ. (A) The role of ISP-I in PD-L1 protein levels was measured through Western blotting. (B) qRT-PCR quantification of *CD274* mRNA expression at gradient ISP-I concentrations (*n* = 3). (C) Immunofluorescence staining examined PD-L1 expression of GBM cells following ISP-I treatment for 24 h. Scale bars = 20 μm. (D) Schematic diagram of implantation, luciferase imaging, and ISP-I and/or TMZ administration. Mice received gavage administration of DMSO, ISP-I (50 mg/kg), TMZ (5 mg/kg), and ISP-I (50 mg/kg) + TMZ (5 mg/kg) daily for 4 weeks (*n* = 8). (E) Bioluminescence imaging was performed to monitor tumor growth in representative mice from each treatment group. (F) Quantification of bioluminescence intensity at postimplantation days 7, 14, 21, and 28 (*n* = 8). (G) Kaplan–Meier survival curves were generated for each treatment group (*n* = 8). (H) Representative HE-stained coronal mouse brain sections among treatment groups. Scale bars = 1 mm. IHC staining quantified Ki-67, γ-H2AX, and PD-L1 expression in tumor specimens. Scale bars = 20 μm. Data: mean ± SD; significance was calculated with one-way ANOVA test. *P < 0.05, **P < 0.01, ***P < 0.001.

ISP-I mediated dose- and time-dependent suppression of PD-L1 protein and *CD274* (PD-L1 gene) mRNA levels (Fig. 3A and B). Confocal microscopy revealed concurrent reductions in PD-L1 expression and GBM cell numbers with escalating ISP-I concentrations (Fig. 3C). Cytotoxicity assays showed that the addition of ISP-I (6 μM) had a minimal destructive effect on bEnd.3 cells and C8-D1A cells (Fig. S2A). A standardized in vitro BBB model using bEnd.3 and C8-D1A cells (Fig. S2B) [37] demonstrated that ISP-I could penetrate the BBB (Fig. S2C). Subsequently, an orthotopic GBM rodent model of GL261-luc was constructed (Fig. 3D). Results showed that both ISP-I and TMZ monotherapy reduced tumor growth, with TMZ monotherapy exhibiting superior efficacy compared to ISP-I alone. However, the combination therapy achieved the lowest tumor burden (Fig. 3E and F) and prolonged median survival time (Fig. 3G). Histological analysis revealed that ISP-I monotherapy was less effective than TMZ at suppressing Ki-67 proliferation indices and inducing DNA damage, but more effective at inhibiting PD-L1 expression. Notably, combination therapy enhanced antitumor efficacy and further increased DNA damage (γ-H2AX) beyond monotherapy effects (Fig. 3H). Throughout treatment, mouse body weight monitoring (Fig. S3A) and hematoxylin and eosin (HE) staining of major organs (Fig. S3B) revealed no notable treatment-related toxicity or pathological alterations with therapeutic ISP-I doses.

### ISP-I induces ICD and activates immune responses in tumor-bearing mice

**Fig. 4.**
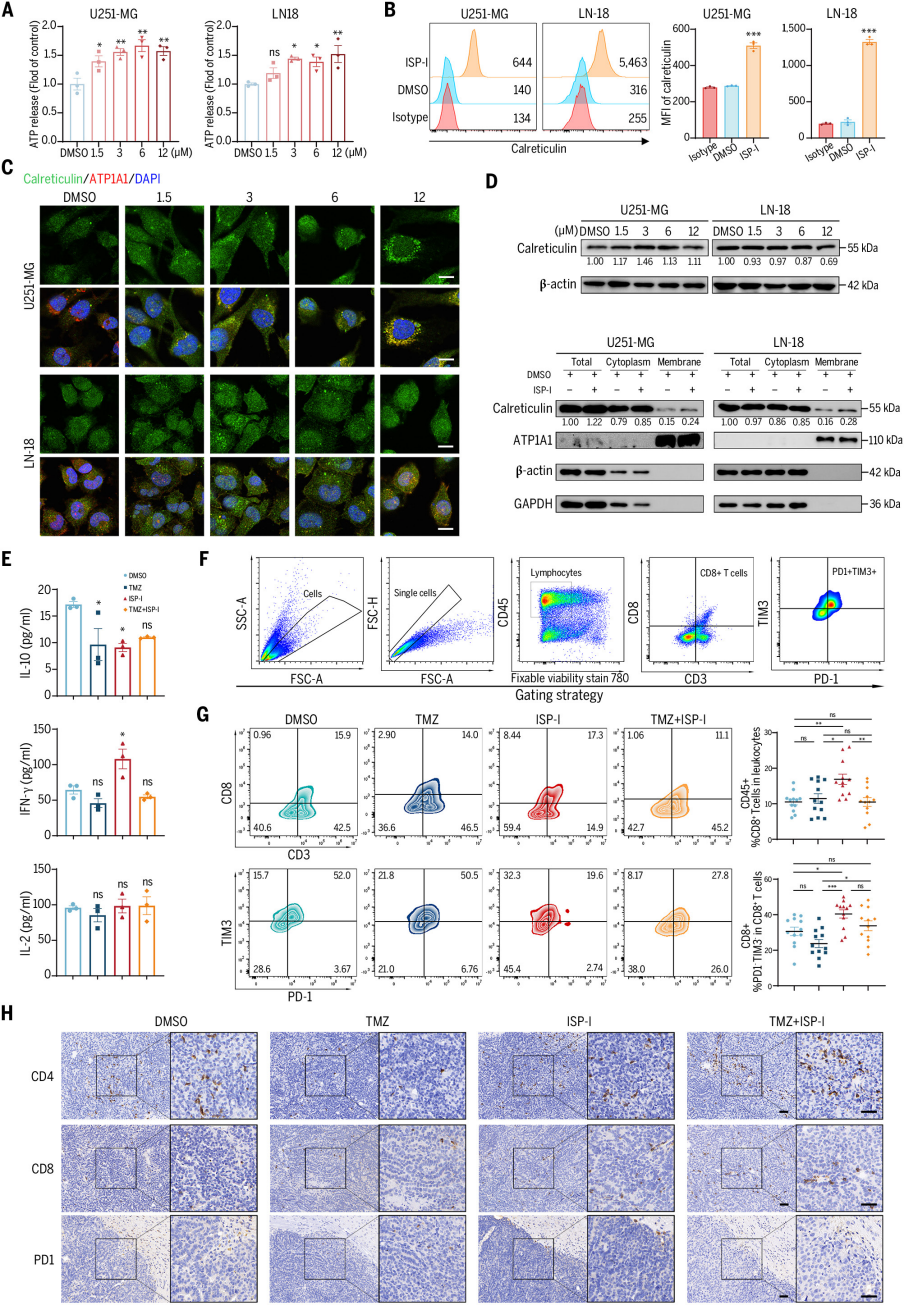
ISP-I induces immunogenic cell death (ICD) and activates immune responses in tumor-bearing mice. (A) ISP-I induced ATP release in U251-MG and LN-18 cells after 24-h treatment. Extracellular ATP secretion was measured using a chemiluminescence assay (*n* = 3). (B) Surface exposure of calreticulin (CRT) on GBM cells, which was detected by flow cytometry after ISP-I treatment at IC_50_ (*n* = 3). (C) Immunofluorescence analysis was performed on cells stained with anti-CRT antibody and 4′,6-diamidino-2-phenylindole (DAPI). (D) Cell lysates were collected to analyze the expression of CRT in the cytoplasmic fraction and cell membrane. (E) An orthotopic glioblastoma xenograft model was established. The concentrations of IL-2, IFN-γ, and IL-10 in serum were evaluated by ELISA on day 28 (*n* = 3). (F) Gating strategies were employed in flow cytometry to analyze exhausted CD8^+^ T-cell subsets (CD45^+^ CD3^+^ CD8^+^ PD-1^+^ TIM3^+^) and effector CD8^+^ T-cell subsets (CD45^+^ CD3^+^ CD8^+^ PD-1^−^ TIM3^−^) in GBM tissues harvested from tumor-bearing mice. (G) Representative flow cytometry plots of tumor-infiltrating CD8^+^ T cells, effector CD8^+^ T-cell subsets, and exhausted CD8^+^ T-cell subsets in tumor tissues from each group (left). Quantification of CD8^+^ and effector CD8^+^ T-cell subsets in each group of mice (right, *n* = 12). (H) Representative IHC staining images for CD4^+^, CD8^+^, and PD-1^+^ cells in tumor tissues. Scale bars = 20 μm. Data: mean ± SD; significance was calculated with one-way ANOVA test. *P < 0.05; **P < 0.01; ***P < 0.001; ns, not significant.

GBM cells were treated with ISP-I for 24 h, after which culture supernatants were collected. As illustrated in Fig. 4A, ISP-I induced elevated extracellular adenosine triphosphate (ATP) levels. Compared to the control group, ISP-I markedly increased calreticulin (CRT) surface exposure on GBM cell membranes, as demonstrated by flow cytometry (Fig. 4B). Immunofluorescence staining demonstrated that CRT expression on the GBM cell membrane was markedly increased following ISP-I treatment (Fig. 4C), which is in accordance with flow cytometry. Western blotting revealed that total CRT expression did not exhibit a notable increase; however, membrane translocation of CRT was observed, reflecting its transfer from the cytoplasm to the cell membrane (Fig. 4D). Enzyme-linked immunosorbent assay (ELISA) analysis (Fig. 4E) for serum cytokines interleukin-2 (IL-2), IL-10, and interferon-γ (IFN-γ) revealed that ISP-I activates the immune system, with increased production of IFN-γ and decreased IL-10.

Given the impact of ISP-I on PD-L1, we specifically investigated the CD8^+^ T-cell subset (Fig. 4F). Compared with the DMSO group, ISP-I significantly enhanced the ratio of CD8^+^ tumor-infiltrating T cells within the tumor bed; however, this effect was abrogated in the ISP-I + TMZ combination group (Fig. 4G). Specifically, treatment with ISP-I resulted in a markedly higher frequency of effector CD8^+^ T cells (PD-1^−^TIM3^−^ subset), and this frequency decreased after TMZ treatment. ISP-I treatment reduced the population of exhausted T cells (PD-1^+^TIM3^+^ subset) in the tumor after TMZ treatment (Fig. S4). Furthermore, immunohistochemistry (IHC) analysis demonstrated that the ratio of CD4^+^ T cells was reduced in TMZ-treated tumor sections, whereas the ISP-I-treated group exhibited an opposite effect (Fig. 4H). In GBM tissues, the baseline CD8^+^ T lymphocyte infiltration was low; in contrast, ISP-I increased intratumoral CD8^+^ T-cell recruitment, a finding validated by flow cytometry (Fig. 4G). Furthermore, IHC analysis demonstrated that PD-1^+^ cells localized primarily at the tumor periphery, with low frequency and no notable intergroup differences (Fig. 4H).

### ISP-I targets FZD5 to inhibit the Wnt/β-catenin signaling pathway

**Fig. 5.**
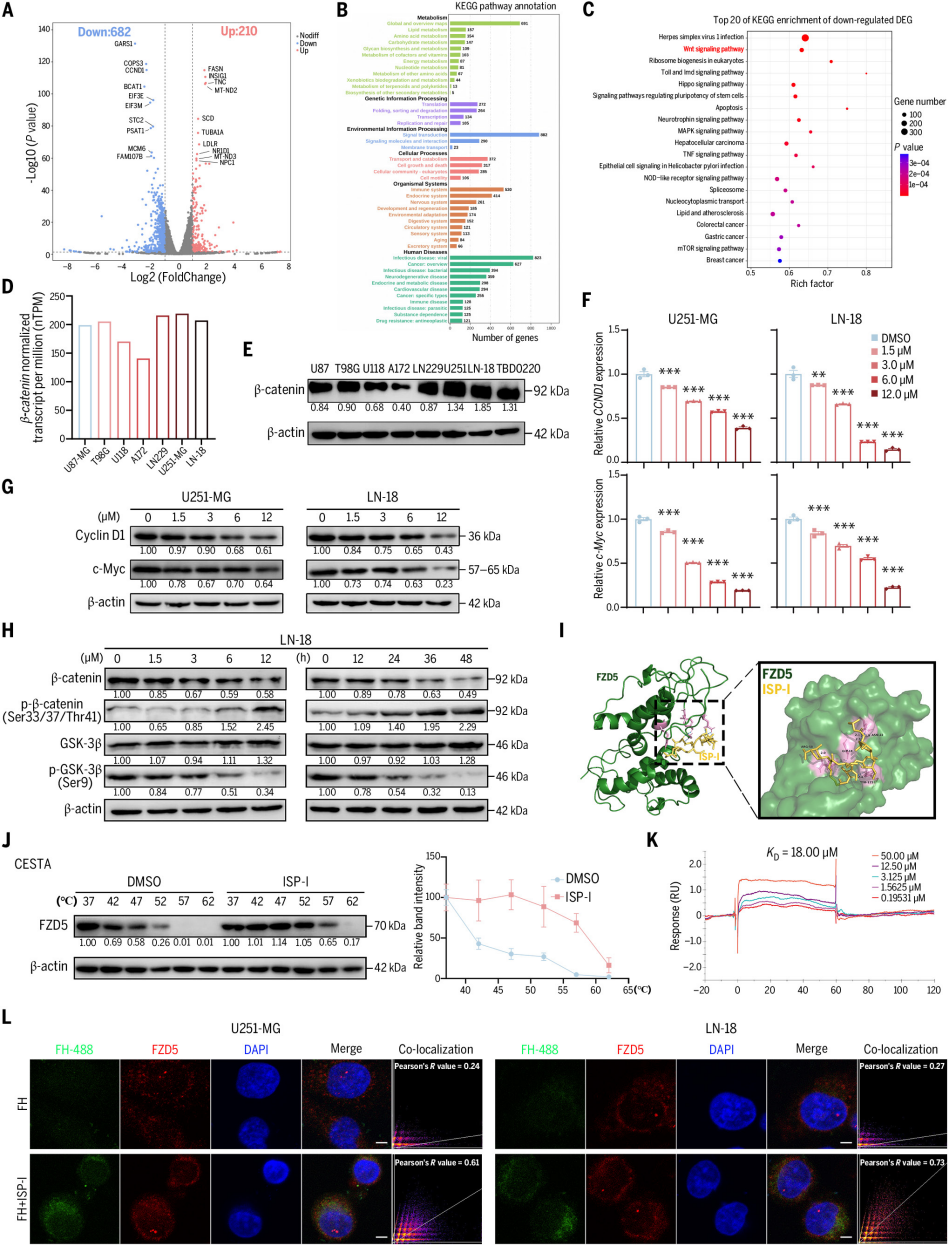
ISP-I targets FZD5 to inhibit the Wnt/β-catenin signaling pathway. (A) RNA-seq analysis indicated that ISP-I alters the transcriptome of LN-18. Volcano plot depicting the distribution and top 10 differentially expressed genes. (B) KEGG pathway enrichment analysis revealed classification and functional annotation of enriched pathways. The horizontal axis represented the number of genes, and the vertical axis showed the classification entries. (C) KEGG analysis highlighted significant suppression of Wnt/β-catenin signaling among altered pathways. (D) Normalized transcript expression values (nTPM) for *CTNNB1* were calculated in GBM cell lines from the Human Protein Atlas project. (E) Changes in β-catenin levels were determined through Western blotting. (F) qRT-PCR analysis of *CCND1* and *Myc* mRNA levels under ISP-I treatment (*n* = 3). (G) Protein levels of cyclin D1 and c-Myc were measured by Western blotting. (H) The effect of ISP-I on levels of core proteins within the Wnt/β-catenin signaling pathway was measured by Western blotting in LN-18 cells. (I) The result of the molecular docking of ISP-I with FZD5 using Autodock Vina was visualized and simulated by PyMOL software. Interaction mode simulation of ISP-I and FZD5 (FZD5: green, ISP-I: yellow, interface residues: pink). (J) ISP-I increased the FZD5 thermal stability as assessed by temperature-dependent CETSA (*n* = 3). (K) SPR assays were performed to quantify the binding affinity of ISP-I to FZD5. (L) Confocal microscopy images of FZD5 (red) and fluorescein-hydrazide-labeled ISP-I (green). Pearson’s coefficient was calculated from scatterplots (ImageJ). Scale bars = 10 μm. Data: mean ± SD; significance was calculated with one-way ANOVA test. **P < 0.01; ***P < 0.001.

RNA sequencing (RNA-seq) analysis of LN-18 cells before and after ISP-I treatment identified 892 differentially expressed genes (DEGs): 210 up-regulated and 682 down-regulated (Fig. 5A). Gene Ontology (GO) enrichment revealed predominant involvement in biological processes (BP) such as cellular processes, biological processes regulation, and metabolism regulation (Fig. S5A). Kyoto Encyclopedia of Genes and Genomes (KEGG) pathway analysis revealed significant alterations in signal transduction pathways (Fig. 5B), notably causing pronounced down-regulation of the Wnt/β-catenin signaling pathway (Fig. 5C). Given the critical regulatory role of β-catenin within this pathway, data from The Human Protein Atlas revealed that U251-MG, LN-18, and LN229 cells expressed the highest *CTNNB1* (β-catenin) transcript levels (Fig. 5D), a finding confirmed at the protein level (Fig. 5E). Notably, these cell lines exhibited the lowest IC_50_ values (Fig. 1B), suggesting that ISP-I may regulate the Wnt/β-catenin pathway. To verify this hypothesis, we validated ISP-I-mediated regulation of *Myc* and *CCND1* (cyclin D1), as canonical Wnt/β-catenin downstream effectors [38,39]. ISP-I dose- and time-dependently inhibited both mRNA and protein expression, mirroring PD-L1 and MGMT regulation (Fig. 5F and G and Fig. S5B). Mechanistically, total β-catenin protein decreased due to reduced GSK-3β phosphorylation at Ser9 (inhibitory site), which enhanced GSK-3β kinase activity, increased β-catenin phosphorylation, and promoted its degradation (Fig. 5H and Fig. S5C).

Molecular docking simulations between ISP-I and 31 Wnt/β-catenin upstream targets (including LRP5/6, FZD1-10, and 19 Wnt ligands) showed the strongest binding affinity for ISP-I-FZD5 interactions (Table S6). The amino acid binding sites are within the FZD5’s cysteine-rich domain (CRD) (Fig. 5I). Consistently, ISP-I potently suppressed FZD5 protein levels among the top candidates (Fig. S5D). Survival and correlation analyses further validated FZD5 as a therapeutic target, showing positive correlations between *FZD5* expression and *CTNNB1*, *CD274*, and *MGMT* levels (Fig. S5E). Therefore, ISP-I may target FZD5 to affect the transcriptional regulation of PD-L1 and MGMT.

As illustrated in the cellular thermal shift assay (CETSA), the extent of protein denaturation in the DMSO group escalated proportionally with rising temperatures (Fig. 5J). ISP-I binding induced a dose-responsive stabilization of FZD5. The isothermal dose-response fingerprint (ITDRF_CETSA_) demonstrated that as ISP-I increased, the thermal stability of FZD5 was gradually enhanced (Fig. S5F). The surface plasmon resonance (SPR) analysis quantified dose-dependent binding of ISP-I to FZD5 protein, using FZD5 antibody binding as a positive control (Fig. S5G). The measured binding affinity (*K*_D_ value) was 18.00 μM (Fig. 5K). To further validate FZD5 as a direct target of ISP-I, we employed fluorescein hydrazide-labeled ISP-I to investigate the colocalization relationship. Confocal microscopy revealed that enhanced membrane adhesion of ISP-I notably increased colocalization with FZD5 (Fig. 5L).

### ISP-I inhibits the transcriptional initiation of *CD274* and *MGMT* via suppressing of β-catenin translocation into the nucleus

**Fig. 6.**
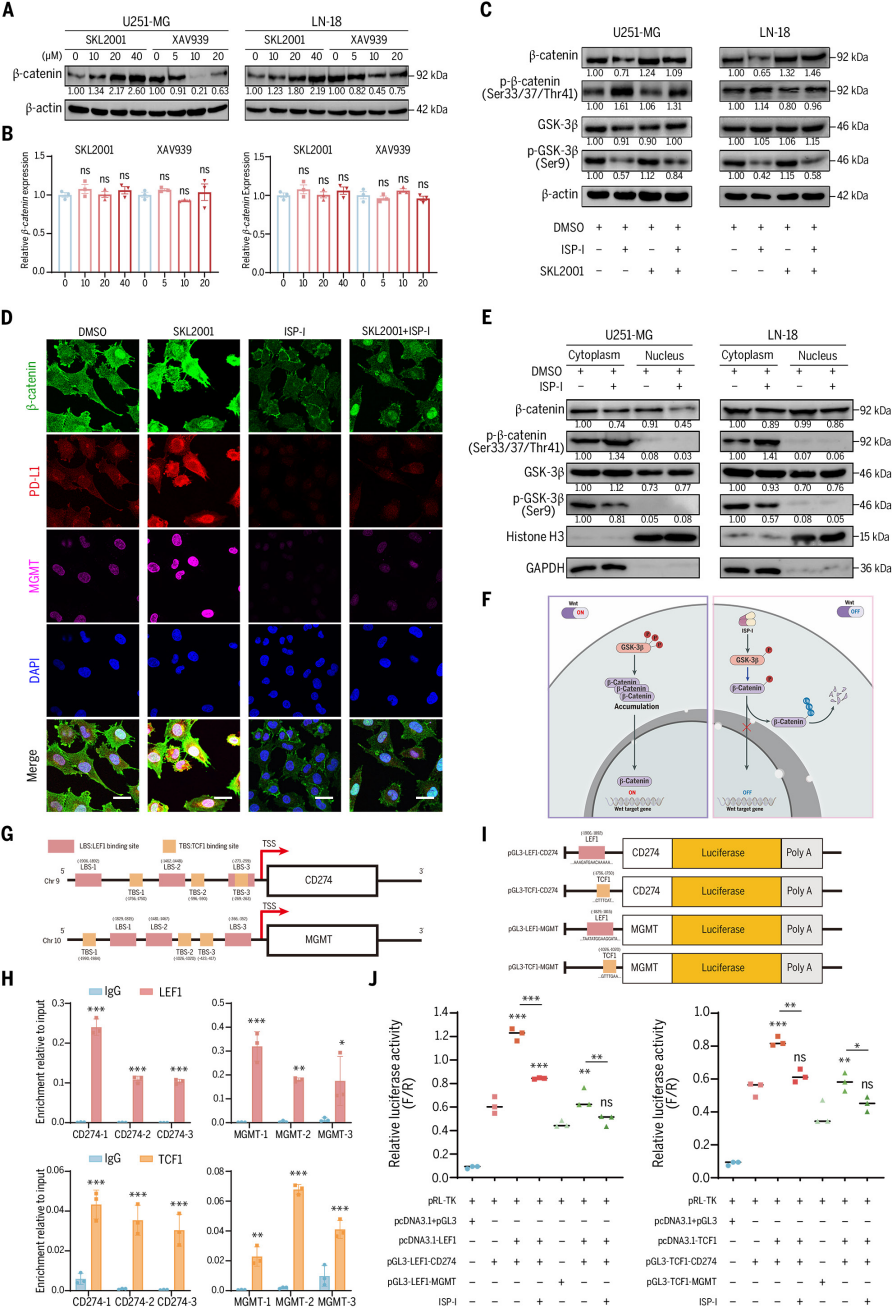
ISP-I inhibits the transcriptional initiation of *CD274* and *MGMT* via suppression of β-catenin translocation into the nucleus. (A and B) The optimal concentration of SKL2001 or XAV939 was verified and determined (*n* = 3). (C) ISP-I reversed the activation of the Wnt/β-catenin pathway by SKL2001. (D) Expression of β-catenin, PD-L1, and MGMT in LN18 cells following 6.0 μM ISP-I in the presence or absence of β-catenin-specific agonist was examined by multiple immunofluorescence. Scale bars = 20 μm. (E) β-Catenin expression in the cytoplasm and nucleus was measured by Western blotting following 6.0 μM ISP-I treatment. (F) Schematic illustration of ISP-I-mediated β-catenin degradation in the cytoplasm by inhibiting GSK-3β phosphorylation, thereby blocking β-catenin nuclear translocation. (G) Schematic illustration of 3 predicted TCF/LEF binding sites in *CD274* and *MGMT* promoters (based on prediction scores). (H) ChIP-qPCR was performed to examine the binding of LEF1 and TCF1 to promoters of *CD274* and *MGMT* (*n* = 3). (I) Schematic representation of selected luciferase plasmid vectors of *CD274* and *MGMT*. (J) Luciferase reporter assay proved that ISP-I negatively regulates transcription of *CD274* and *MGMT* genes (*n* = 3). Data: mean ± SD; significance was calculated with one-way ANOVA test. *P < 0.05; **P < 0.01; ***P < 0.001; ns, not significant.

The optimal concentrations of SKL2001 (a β-catenin agonist) and XAV939 (a β-catenin inhibitor) were determined (Fig. 6A and B). ISP-I reversed SKL2001-induced pathway activation (Fig. 6C). Analysis of β-catenin subcellular distribution revealed that ISP-I curtailed β-catenin accumulation in both the cytoplasmic and nuclear compartments. This suppression correlated with alterations in PD-L1 and MGMT levels (Fig. 6D). Similarly, the accumulation of β-catenin declined in GBM cells following ISP-I treatment, especially in the nucleus (Fig. 6E). Alterations in p-β-catenin and p-GSK-3β mirrored total protein trends, indicating that ISP-I blocks β-catenin nuclear translocation to suppress Wnt/β-catenin signaling (Fig. 6F).

To ascertain whether ISP-I regulates the transcriptional regulation of *CD274* and *MGMT* through the Wnt/β-catenin pathway, we performed chromatin immunoprecipitation-quantitative polymerase chain reaction (ChIP-qPCR) to assess T-cell factor/lymphoid enhancer factor (TCF/LEF) binding to their promoters. The JASPAR database was used to assess the likelihood of binding between the TCF/LEF family (LEF1, TCF1, TCF3, and TCF4) [40] and the promoter regions of *CD274* and *MGMT* (Table S7). The 2 transcription factors with the highest probabilities, TCF1 and LEF1, were identified as the subjects for subsequent research (Fig. 6G). ChIP-qPCR confirmed TCF1/LEF1 binding (Fig. 6H), and the promoter fragments with the most significant TCF/LEF binding differences were selected for analysis (Fig. 6I). Dual-luciferase reporter assays revealed that TCF1/LEF1 overexpression significantly enhanced *CD274* and *MGMT* promoter activity. By contrast, ISP-I treatment markedly suppressed this transactivation effect (Fig. 6J).

### ISP-I inhibits β-catenin to regulate PD-L1 and MGMT expression in tumor-bearing mice

**Fig. 7.**
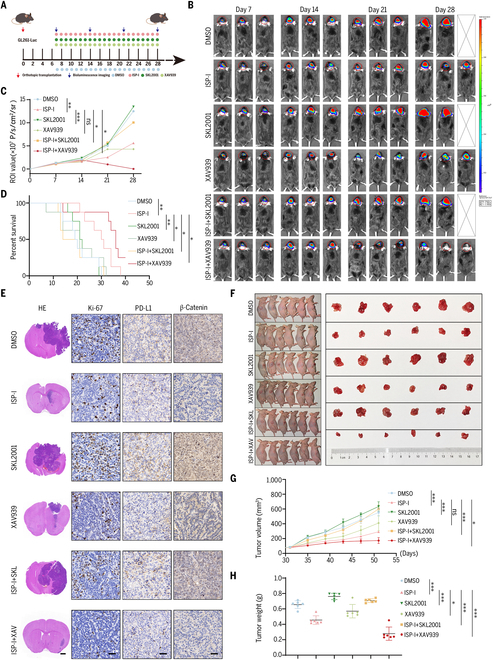
ISP-I inhibits β-catenin to regulate PD-L1 and MGMT expression in tumor-bearing mice. (A) Schematic of experimental treatment of GL261-luc orthotopic model. Mice (*n* = 8) were treated with DMSO, ISP-I (50 mg/kg), SKL2001 (10 mg/kg), XAV939 (10 mg/kg), ISP-I (50 mg/kg) + SKL2001 (10 mg/kg), or ISP-I (50 mg/kg) + XAV939 (10 mg/kg) daily via oral gavage for 21 days. (B) Assessment of tumor growth in each group was qualified by bioluminescence imaging. (C) Bioluminescence intensity (BLI) was quantified on days 7, 14, 21, and 28 postimplantations to monitor growth dynamics (*n* = 8). (D) Kaplan–Meier survival curves were constructed to compare overall survival across groups (*n* = 8). (E) Representative HE staining images of mouse cerebral tissues from each group, depicting tumor volumes. Left, scale bars = 1 mm. Immunohistochemical staining for Ki-67, PD-L1, and β-catenin in tumor tissues. Right, scale bars = 20 μm. (F) Subcutaneous tumor-bearing nude mice (LN-18 cells) were treated with vehicle (DMSO), ISP-I, SKL2001, XAV939, ISP-I + SKL2001, and ISP-I + XAV939 by gavage for 3 weeks, respectively (*n* = 6). (G) Measure the tumor size every 4 days after treatment. Volume = 0.52 × length × width^2^ (*n* = 6). (H) The wet weight of collected tumors from each group (*n* = 6). Data: mean ± SD; significance was calculated with one-way ANOVA test. *P < 0.05; **P < 0.01; ***P < 0.001; ns, not significant.

In an orthotopic intracranial GBM mouse model (Fig. 7A), bioluminescence imaging (BLI) revealed accelerated tumor growth in SKL2001-treated mice compared with the DMSO control group. Both ISP-I and XAV939 monotherapies effectively suppressed tumor hyperproliferation, while their combination achieved the maximal reduction in tumor burden (Fig. 7B and C). Survival analysis showed significantly shorter survival in the DMSO and SKL2001 groups. Although ISP-I monotherapy outperformed XAV939 alone, the ISP-I + XAV939 combination extended median survival most substantially (Fig. 7D). HE staining showed that SKL2001 treatment promoted tumor overgrowth, leading to a notable midline shift in the brain. By contrast, the ISP-I + XAV939 combination notably reduced tumor volume compared with all other groups. IHC analysis results indicated that the group treated with both ISP-I and XAV939 showed a markedly diminished rate of tumor growth (as assessed by Ki-67). ISP-I treatment decreased β-catenin expression and concomitantly down-regulated PD-L1. By contrast, SKL2001 treatment induced marked up-regulation of both PD-L1 and β-catenin, a phenotype that was effectively reversed by ISP-I (Fig. 7E), suggesting a potential regulatory link between β-catenin and PD-L1 expression.

We established a subcutaneous xenograft model in mice using LN-18 cells and grouped and treated the mice as described above. Compared with the control group, tumor size in the SKL2001 group exhibited a notable increase, whereas both ISP-I and XAV939 treatments effectively constrained tumorigenesis—with ISP-I showing superior efficacy to XAV939. The ISP-I + XAV939 combination produced the strongest antitumor effect (Fig. 7F), consistent with tumor volume growth and weight measurements (Fig. 7G and H). Mice were euthanized and tumor samples were harvested for IHC analysis to examine Ki-67, PD-L1, MGMT, and β-catenin expression (Fig. S6). Alterations in PD-L1 and MGMT expression in tumor tissue paralleled those of β-catenin, suggesting coordinated regulation.

## Discussion

TMZ is the sole chemotherapeutic agent proven to extend the overall survival of GBM patients [2,3]. However, the development of novel drugs faces challenges including lengthy timelines, high costs, elevated risk, uncertain efficacy, and inherent resistance. Repurposing clinically used or trial-stage drugs has emerged as a promising solution to these challenges [41–43]. ISP-I represents a repurposing candidate due to its antineoplastic activity and circumvention of traditional development barriers [35,36]. ISP-I has been found to induce cancer cell-specific genomic instability [36]. Mechanistically, ISP-I induces DNA double-strand breaks to exert cytotoxicity, and its synergistic enhancement of DNA damage with TMZ provides a rationale for combined therapy. Notably, the molecular basis of this interaction in DNA damage regulation requires further exploration. Besides, U251-MG and LN-18 cells exhibited the highest sensitivity to ISP-I, while showing markedly divergent TMZ sensitivity, which strengthened the validity of combined therapy evaluation and established an ideal model for synergistic studies.

Given the pivotal role of MGMT in TMZ sensitivity, therapeutic strategies targeting its expression are attractive [3,5]. Here, we demonstrated that ISP-I exhibits potent anti-GBM activity, particularly in synergy with TMZ. Mechanistically, ISP-I suppresses MGMT expression, thereby reversing MGMT-dependent TMZ resistance and enhancing chemosensitivity. Notably, we faced challenges due to the inability of GL261 cells to express MGMT protein. Our initial attempts to construct an intracranial tumor model using LN-18 or T98G cells to verify ISP-I’s reduction of MGMT expression in vivo were unsuccessful, due to tumor microenvironment (TME) complexity. Consequently, we utilized subcutaneous xenograft models for in vivo experiments.

The brain harbors an immunosuppressive microenvironment, with GBM characterized by scarce immune cell infiltration, classifying it as a “cold” tumor with limited response to immune checkpoint inhibitors (ICIs) [10]. Systemic TMZ administration exacerbates immune dysfunction in GBM patients by inducing lymphopenia and T-cell exhaustion [44,45], driving tumor immune evasion. Although ICIs can reinvigorate systemic immunity [46,47], their efficacy in GBM is constrained by poor BBB penetration [48]. ICD, characterized by the release of damage-associated molecular patterns (DAMPs), is induced by anticancer therapies and relies on TME conditions to activate adaptive T-cell responses [49,50]. ATP is secreted during the premortem phase of ICD, and surface exposure of CRT serves as a positive signal to promote immune activation [49,51]. Our study showed that ISP-I treatment induces ICD and alters PD-L1, IL-10, and IFN-γ levels, implicating CD8^+^ T cells in its immunomodulatory effect. Contrastingly, TMZ alone did not alter tumor-infiltrating lymphocytes, but blunted ISP-I-induced expansion of effector CD8^+^ T cells. Conversely, ISP-I counteracted TMZ-mediated immunosuppression. Notably, although CD8^+^ T cells serve as key effector cells in antitumor immunity, the GBM microenvironment is dominated by immunosuppressive cell populations (e.g., tumor-associated macrophages, microglia, and myeloid-derived suppressor cells) [10,52]. The dynamics of these cells were not quantified in this study, which may limit our understanding of ISP-I’s immunomodulatory role. Tumor PD-L1 levels are a key biomarker for ICI efficacy [53,54], yet its role in GBM remains undefined. Theoretically, ISP-I-mediated PD-L1 down-regulation may hinder the efficacy of anti-PD-L1 agents, as reduced PD-L1 expression could attenuate checkpoint blockade. Currently, since ICIs have shown unsatisfactory efficacy in GBM clinical trials, the scientific rationale for combining ISP-I with ICIs requires further validation.

Macrolide antibiotics disrupt the Wnt/β-catenin signaling pathway [55,56], and here we show that ISP-I enhances β-catenin phosphorylation, promoting cytoplasmic degradation and reducing nuclear accumulation. This blocks TCF1/LEF1 binding to *CD274* and *MGMT* promoters. Within the Wnt/β-catenin signaling pathway, the extracellular N-terminus of FZDs contains a CRD that serves as the primary structural module for Wnt ligand binding [19,40]. Notably, our results indicate that ISP-I binds to the FZD5-CRD region [57]. FZD5 serves as a signaling receptor for Wnt5A and Wnt7A/B [58,59], with Wnt5A exhibiting a negative correlation with GBM prognosis [60]. Furthermore, targeting Wnt7A/B has been demonstrated to enhance responses to TMZ [61]. ISP-I-FZD5 binding disrupts receptor conformational stability, enhancing FZD5 degradation and competitively inhibiting binding affinity for cognate ligands such as Wnt5A and Wnt7A/B. These findings confirm that ISP-I inhibits PD-L1 and MGMT expression by directly targeting FZD5 to regulate the Wnt/β-catenin signaling pathway.

However, there are still certain limitations. The in vitro BBB cannot fully mimic the complete structure under in vivo conditions, and the impact of the blood–tumor barrier on ISP-I delivery remains undefined. Lack of real-time imaging techniques hindered quantification of ISP-I distribution in cerebrospinal fluid and tumors. Additionally, as an active ingredient of macrolide antibiotics, the efficacy, dosage, and treatment course of ISP-I require reevaluation. Further experiments are also required to clarify the specific role of the target FZD5 in GBM drug resistance and immune regulation. These considerations also limit its potential for rapid application in treating other diseases.

## Conclusion

**Fig. 8.**
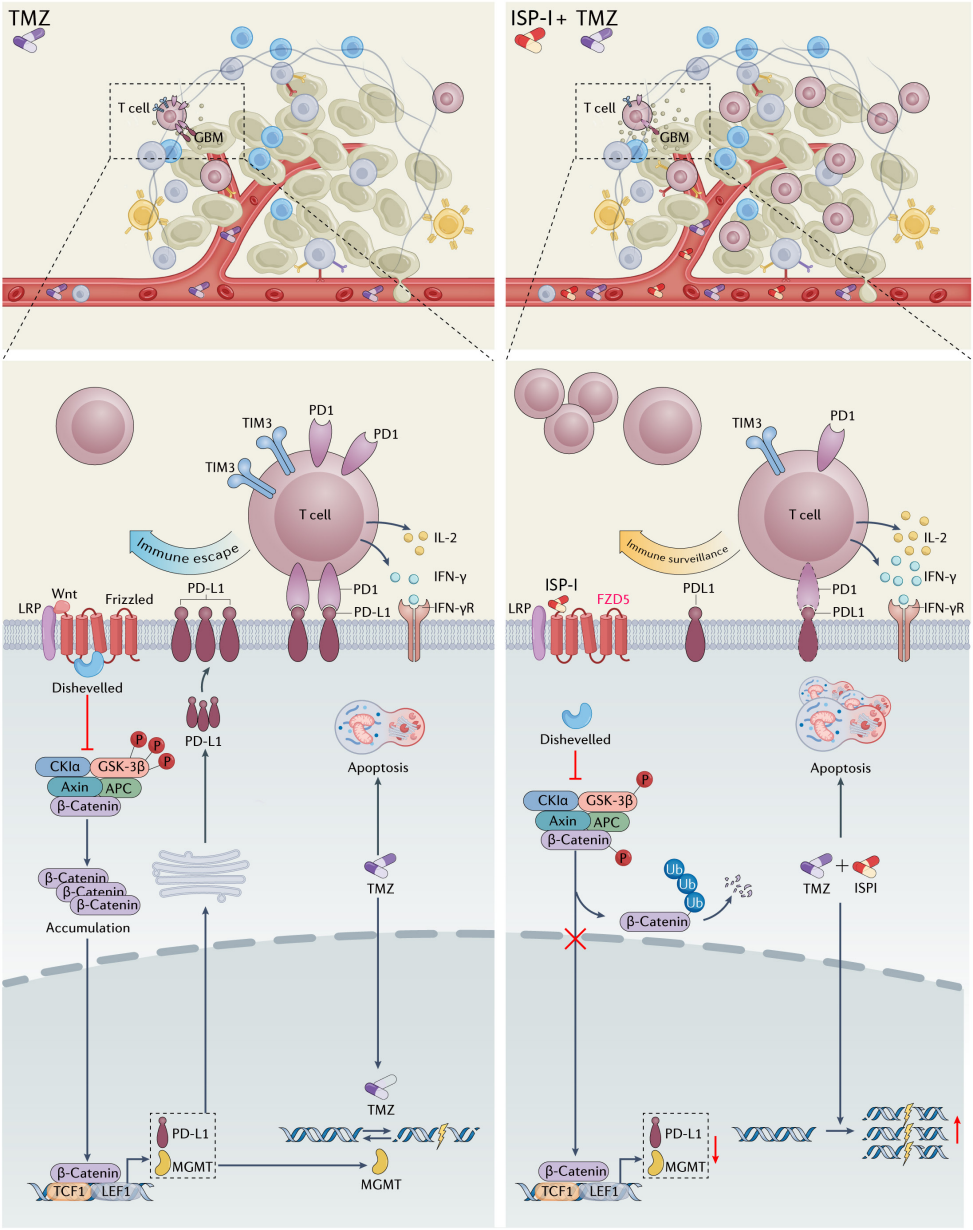
ISP-I targets FZD5, thereby inhibiting the Wnt/β-catenin signaling pathway. This inhibition leads to the suppression of PD-L1 and MGMT expression, augmenting glioblastoma cell susceptibility to TMZ and reprogramming the immunosuppressive microenvironment.

ISP-I synergizes with TMZ to enhance anti-GBM efficacy by targeting the FZD5/Wnt/β-catenin pathway, concurrently suppressing MGMT and PD-L1 expression. It is particularly suitable for GBM tumors characterized by MGMT-overexpressing and TMZ-induced immunosuppressive microenvironments (Fig. 8). These findings offer critical evidence for clinical translation and rational combination therapeutic strategies.

## Materials and Methods

### Compounds and reagents

ISP-I was synthesized by Shanghai Tonglian Pharmaceutical Co., Ltd. ISP-I was prepared as a stock solution (100 mM) in DMSO (D2650, Sigma, Shanghai, China) and then further diluted to suitable concentrations using the cell culture medium (the final DMSO concentration in the culture medium maintained below 0.015%) for in vitro experiments. TMZ (HY-17364), SKL2001 (HY-101085), XAV939 (HY-15147), and fluorescein hydrazide (HY-151644) were procured from MedChem Express (Shanghai, China). In the case of animal experiments, the reagents were dissolved in a mixture of DMSO, polyethylene glycol 300 (PEG-300, IP9020, Solarbio, Beijing, China), Tween-80 (BS118, Solarbio, Beijing, China), and saline at appropriate concentrations at a volume ratio of 2:8:1:9.

### Cell culture and cell viability assay

Cell lines U87-MG, T98G, U118, A172, TBD0220, LN229, U251-MG, GL261, LN-18, bEnd.3, and C8-D1A were obtained from the Cell Bank of the Chinese Academy of Sciences. Cells were cultured in Dulbecco’s Modified Eagle Medium (DMEM) containing 10% fetal bovine serum (FBS; Gibco, Waltham, MA, USA) under 5% CO_2_ at 37 °C. Cell viability was assessed by seeding 5,000 cells per well into 96-well plates and culturing them for 24 h. The dose of ISP-I was 6.0 μM for in vitro time-course experiments. In the cell experiments of the combined evaluation, the U251-MG cell line was treated with ISP-I (8.0 μM) or TMZ (80.0 μM), and the LN-18 cell line was treated with ISP-I (5.0 μM) or TMZ (300.0 μM). After administering the selected drugs at specified concentrations and time points, the medium was replaced with fresh culture medium. The cell viability was evaluated using the CCK-8 assay (CK04, Dojindo, Kumamoto, Japan) following a 60-min incubation period at 37 °C. Absorbance was measured at 450 nm using an optical density reader (BioTek, VT, USA). The CI was determined using Compusyn software: CI > 1 indicates antagonism, CI < 1 signifies synergy, and CI = 1 denotes an additive effect [62].

### EdU and TUNEL assay

EdU assays were conducted using an EdU kit (KGA335, KeyGen, Nanjing, China). First, 96-well plates were inoculated with 4 × 10^4^ cells per well for drug treatment. Subsequently, EdU working solution (1:1,000) was added and incubated for 2 h at 37 °C. After incubation, cells were fixed with 4% paraformaldehyde for 30 min at room temperature, permeabilized with 0.5% Triton X-100 for 20 min. In accordance with the manufacturer’s protocol, click reaction solution was incubated with the cells for 30 min in the dark before adding the Hoechst solution. Similarly, the TUNEL detection kit (KGA7072, KeyGen, Nanjing, China) was employed following the manufacturer’s protocol to detect cell DNA damage. Fluorescence images of groups were obtained utilizing a Nikon TI2-E fluorescence microscope (Nikon, Japan).

### Apoptosis flow cytometry analysis

Detection was conducted 24 h after the administration of ISP-I and/or TMZ. The instructions of the Annexin V/PI detection kit (KGA107, KeyGen, Nanjing, China) were strictly followed, and the cells were treated with the kit’s reagents. Apoptotic cell proportions were quantified by flow cytometry using a BD Verse cytometer (BD Biosciences, CA, USA).

### Protein extraction and Western blotting analysis

A cytosolic cell lysis kit (KGB5300, KeyGen, Nanjing, China) and radio immunoprecipitation assay lysis buffer (P0013B, Beyotime, Shanghai, China) were used to isolate nuclear, cytosolic, and membrane proteins. Following the isolation of proteins from the cells, a bicinchoninic acid assay assay kit (WB6501, NCM, Suzhou, China) was used to quantify the sample protein concentrations. Protein lysates were subjected to sodium dodecyl sulfate-polyacrylamide gel electrophoresis and transferred onto polyvinylidene fluoride (IPVH00010, Millipore, China). Membranes were blocked with 5% skimmed milk for at least 1 h at room ambient temperature. After overnight incubation with the primary antibody, the membranes were incubated with horseradish peroxidase-conjugated secondary antibody (CST, Danvers, MA, USA) for 1 h. Immunoreactive bands were imaged using an ECL detection system (WBKLS0500, Millipore, Shanghai, China) and ImageQuantLAS500 chemiluminescence apparatus (GE, USA). Differences in protein expression were assessed using the ImageJ software (National Institutes of Health). β-Actin was used as the loading control. Table S1 provides a comprehensive list of the antibodies.

### RNA-seq and data analysis

RNA-seq libraries were processed on the NovaSeq 6000 platform (Illumina) by Shanghai Personal Biotechnology Co., Ltd. Differential gene expression (DEG) analysis was performed using DESeq2 (v1.38.3) with stringent thresholds: |log2FoldChange| > 1, adjusted *P* value < 0.05 to identify genes with significant differences. To assess enrichment of biological processes, molecular functions, and cellular components, GO enrichment analysis was performed using ClusterProfiler (v4.6.0). Significance was determined by hypergeometric testing (*P* < 0.05), with separate analyses for all DEGs, up-regulated DEGs, and down-regulated DEGs to identify directionality-specific functional associations. ClusterProfiler (version 4.6.0) was used to perform KEGG pathway enrichment analysis on DEGs.

### Quantitative real-time polymerase chain reaction

Total RNA was extracted using TRIzol (Takara, Shiga, Japan) and cDNA was synthesized using Evo M-MLV RT Master Mix (AG11706, AG Bio, Changsha, China). Quantitative real-time polymerase chain reaction (qRT-PCR) was performed on a 7500 Fast Real-Time PCR System (Applied Bio, CA, USA) using SYBR Green PCR Master Mix (K1070, APE Bio, Houston, TX, USA). The endogenous normalization control employed in this study was glyceraldehyde-3-phosphate dehydrogenase (GAPDH). Primer sequences required for the experiment are provided in Table S2. Relative gene expression was quantified via the 2^−ΔΔCt^ method using cycle threshold (Ct) values, with normalization to GAPDH. Results are derived from at least 3 biologically independent experiments.

### Immunofluorescence and confocal imaging

The cells, which were cultivated on confocal plates with varying treatments, were fixed with 4% paraformaldehyde (BL539A; Biosharp, Hefei, China), permeabilized with Triton X-100 (0.5%, Sigma, Shanghai, China), and blocked using 5% bovine serum albumin. Following overnight incubation with the primary antibody at 4 °C, samples were further incubated with a species-specific secondary antibody for 1 h at room temperature. Nuclear counterstaining was conducted using 4′,6-diamidino-2-phenylindole. Intracellular actin was stained with phalloidin. Images of cells were acquired using a laser scanning confocal microscope (Nikon, Japan). Pearson’s colocalization coefficients were calculated using ImageJ software, and the resulting data were plotted in a scatterplot of red and green pixel intensities for quantification. The antibodies used in immunofluorescence are listed in Table S3.

### Mouse models and treatments

Animal studies were approved by the Animal Experiment Committee of Zhujiang Hospital, Southern Medical University (approval no. LAEC-2022-232) and were conducted in strict adherence to guidelines for the care and use. Mice were kept in a controlled environment free from pathogens, with a 12-h light/dark cycle, maintained at ideal temperature and humidity. An in situ GBM rodent model was constructed using 4-week-old male C57BL/6J mice by stereotactically injecting GL261-luciferase cells (5 × 10^4^ cells/animal) into the intracranial space. Four-week-old male BALB/c nude mice were utilized to establish subcutaneous xenograft tumor models. The LN18 cell (1 × 10^7^ cells/animal) suspension and Matrigel Matrix (#356234, Corning, NY, China) were blended in a 1:1 ratio and injected subcutaneously into the right flank. All mice were randomly assigned to each experimental group. Survival times were documented, and mouse brains were obtained for further analysis. The dissolved drugs formulation comprised 45% saline, 40% polyethylene glycol-300, 10% DMSO, and 5% Tween-80. All animal experiments used DMSO at ≤1% v/v (vehicle control). The BLI was performed to monitor the growth of intracranial tumor using an IVIS Spectrum Imaging System (Perkin, Branford, CT, USA).

### Cytokines quantification by ELISA

The mouse IFN-γ (JL10967), IL-2 (JL20256), and IL-10 (JL20242) ELISA kits were procured from Jianglai Biological Technology Co., Ltd. (Shanghai, China). ELISA kits were used to quantify serum cytokine concentrations in accordance with the manufacturer’s protocol. Standard curves were constructed using known concentrations of recombinant cytokines. The concentrations of cytokines in each sample were determined by evaluating them by comparing them against the standard curve.

### Flow cytometry of tumor-infiltrating immune cells

Brain tumors were resected aseptically, weighted and mechanically minced. Single-cell suspensions were generated by enzymatic and mechanical dissociation of tumors using the Brain Tumor Dissociation Kit (Miltenyi Bio, Bergisch Gladbach, Germany) in conjunction with the gentleMACS Octo Dissociator (Miltenyi Bio). Subsequently, the cellular suspension was filtered through a 70-μm cell strainer and resuspended in FACS staining buffer (phosphate-buffered saline with 2% FBS). The lysis of red blood cells was achieved by employing a red blood cell removal solution. Cells were then stained with a panel of cell surface marker-specific antibodies, as detailed in Table S4. Subsequently, the cells resuspended in buffer were analyzed using Beckman CytoFLEX (Brea, CA, USA). Gating was performed using unstained and single-stained cell controls to define gating parameters.

### HE and IHC staining

Tissues embedded in paraffin were sectioned into 4- to 5-μm pieces, dewaxed, and subsequently hydrated. Antigen retrieval was performed using a citrate buffer at 95 °C for a half hour. Samples were incubated with primary antibodies against Ki-67, γ-H2AX, PD-L1, CD4, CD8, PD1, MGMT, and β-catenin overnight at 4 °C. Sections were then incubated with secondary antibodies for 1 h. Sections were visualized using dimethoxybenzidine. Digital images were obtained using the scanner (3DHISTECH, Budapest, Hungary).

### Molecular docking

The chemical structure of ISP-I was retrieved from the PubChem database. The Protein Data Bank and AlphaFold Protein Structure Database were used to obtain the 3-dimensional structures of the core proteins. AutoDock Vina was used for the docking analysis. Prior to analysis, all solvents, water molecules, and cocrystallized ligands were eliminated from the structures. The docking procedure was executed, generating 50 structures per calculation to acquire the optimal binding conformation. PyMOL was used to represent the docking structures as either cartoons or surface models.

### CETSA and ITDRF_CETSA_

GBM cells were incubated with ISP-I (6 μM) for 4 h in CETSA experiments. The DMSO group served as the negative control. Cells were harvested and evenly distributed into 10 PCR tubes, which were heated to 6 temperatures (37 to 62 °C) for 3 min and then incubated at 4 °C for 3 min. Subsequently, samples were lysed for protein extraction. Western blotting was performed to analyze soluble proteins. The procedures used in the ITDRF_CETSA_ experiments were identical to those employed in the CETSA. Cells were incubated with various concentrations of ISP-I (10^−5^ to 30 μM) at 55 °C.

### Surface plasmon resonance

ISP-I concentrations ranging from 0.1953 to 50.00 μM were passed over the CM5 chip surface. FZD5 protein (10 μg/ml, 10473-H08H, Sino Bio, Beijing, China) was loaded at a flow rate (50 μl/min) for 60 s, followed by a dissociation flow period of 30 s at 25 °C. SPR analysis was performed on Biacore T200 (Cytiva, Marlborough, MA, USA) to detect binding interactions between ISP-I and FZD5, with response units recorded in real time. Data were analyzed using the Langmuir binding model, and the equilibrium dissociation constant (*K*_D_) was calculated as the ratio of the dissociation rate constant (*K*_off_) to the association rate constant (*K*_on_), where *K*_D_ = *K*_off_/*K*_on_. An anti-FZD5 antibody (68204-H001, Sino Bio, Beijing, China) was used as a positive control.

### ChIP-qPCR

Assays were conducted using the Agarose ChIP Kit (26156, Thermo Fisher). In brief, chromatin from LN-18 cells was utilized to ascertain the total input DNA and was subsequently incubated with target-specific primary antibodies or normal rabbit IgG overnight. Antibodies against TCF1 (2203, CST) and LEF1 (ab137872, Abcam) were used to immunoprecipitate CD274/MGMT chromatin complexes. Anti-IgG antibody (ab172730, Abcam) was used as a negative control. Immunoprecipitated chromatin was analyzed using a CFX Connect Real-Time PCR Detection System (1855201, Bio-Rad). PCR primer sequences are listed in Table S5.

### Online database analysis

The 3-dimensional molecular structure of ISP-I (CAS: 267662-22-6) was obtained from PubChem (https://pubchem.ncbi.nlm.nih.gov/). The potential proteins were obtained from Protein Data Bank (https://www.rcsb.org/) and AlphaFold database (https://alphafold.com/). Patient survival analysis and gene correlation analysis in gliomas were performed using the Chinese Glioma Genome Atlas (http://www.cgga.org.cn) and GEPIA2 (http://gepia2.cancer-pku.cn). Transcript levels of β-catenin in cell lines were interrogated via the Human Protein Atlas (https://www.proteinatlas.org), which integrates multi-omics data across cell lines and tissues. Transcription factor binding sites were predicted using the JASPAR database (http://jaspar.genereg.net), a widely used resource for eukaryotic cis-regulatory element analysis.

### Dual-luciferase reporter assay

The promoter sequence fragments of the target genes that exhibited the most pronounced differences were selected based on ChIP experiments. These were then subjected to digestion with the restriction endonucleases XhoI (R0146, NEB) and NheI (R3131, NEB), which recognize the C/TCGAG and G/CTAGC sequences, respectively. Primers were ligated into the pGL3-basic plasmid (Promega). The coding sequence regions of LEF1 and TCF1 were cloned into pcDNA3.1. Subsequently, luciferase reporter systems were constructed for the CD274 and MGMT promoters. LN-18 cells were evenly plated into 6-well plates at a density of 1 × 10^6^ cells/well for 16 h. The luciferase reporter plasmid, pcDNA3.1 vector plasmid, and pRL-TK vector plasmid (Promega) were cotransfected separately into each group using Lipofectamine 3000 transfection reagent (L3000015, Thermo Fisher). The final amount of plasmid used (μg/μl) was diluted 2.5 times with Opti-MEM, resulting in a final volume of 0.5 ml. This solution was then added to a 6-well plate and reacted for 6 h to facilitate transfection. Following the removal of the transfection reagent, the experimental group of cells was incubated in DMEM for 48 h. The cells were treated with 6 μM ISP-I for 24 h. Luciferase activity was subsequently quantified using a dual-luciferase reporter assay (E1910, Promega).

### BBB models in vitro

A Transwell chamber (Corning Transwell) was utilized to construct a classical in vitro model mimicking BBB. bEnd.3 cells were inoculated onto the apical membrane of Transwell insert, while C8-D1A cells were seeded onto the basolateral surface. To facilitate astrocyte seeding, the Transwell was inverted to position the basolateral side upward, ensuring proper orientation for coculture setup [37]. The aldehyde and ketone groups of ISP-I were labeled with fluorescein hydrazide (FH excitation 488 nm). Following the placement of ISP-I (6 μM) in the upper compartment at various times, the transport of ISP-I across the BBB was evaluated by incubation with FH (20 μM) for 1 h at 37 °C.

### Statistical analysis

Statistical analyses were conducted using the GraphPad Prism 9 (San Diego, CA, USA). The Student’s *t* test was used to compare the 2 groups. One-way analyses of variance (ANOVAs) were employed to assess differences among 3 or more groups. Correlation between 2 variables was evaluated using the Pearson correlation test depending on the conditions. The prognostic significance of categorical variables was determined using the log-rank test. Error bars indicate the mean ± standard deviation (SD). *P < 0.05; **P < 0.01; ***P < 0.001; ns, not significant.

## Data Availability

The data are freely available upon request.
