# Supplementary material for: Isovalerylspiramycin I Reprograms the Immunosuppressive and Temozolomide-Resistant Microenvironment by Inhibiting the Frizzled-5/Wnt/β-Catenin Pathway in Glioblastoma
Source: Research (Wash D C). 2025 Aug 13;8:0828. doi: 10.34133/research.0828 (PMC12349883; doi:10.34133/research.0828)
Supplement: Supplementary 1 — Figs. S1 to S6 Tables S1 to S7 [file research.0828.f1.docx]

**Supporting Information of**

**Isovalerylspiramycin I Reprograms the Immunosuppressive and Temozolomide-resistant Microenvironment by Inhibiting the FZD5/Wnt/β-catenin Pathway in Glioblastoma.**

**Including:**

**Supplementary figures: Fig.S1 to S6.**

**Supplementary tables: Table S1 to S7.**

**
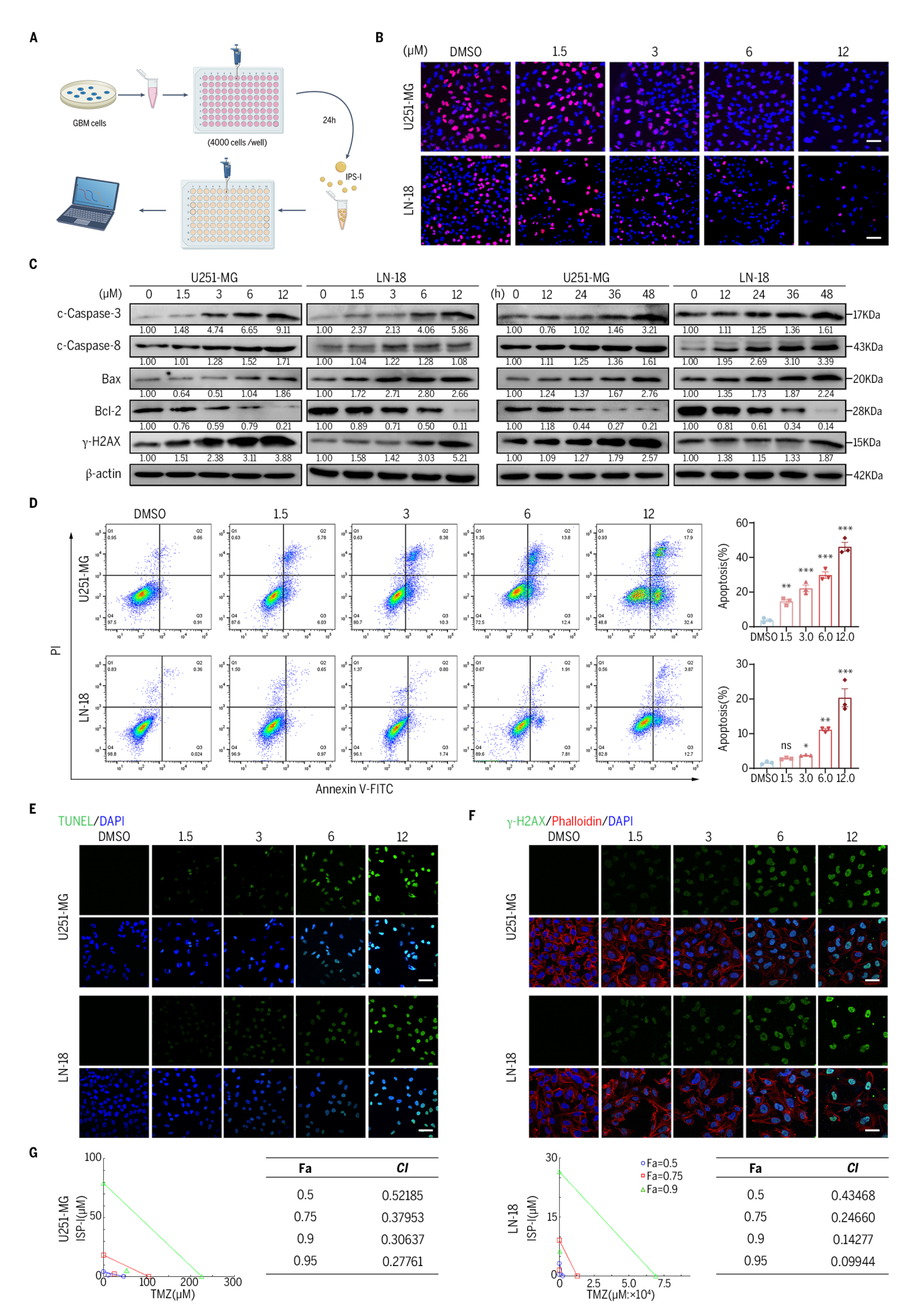
**

**Fig. S1.** ISP-I induces cytotoxicity and apoptosis by modulation of DNA damage in GBM cells. (A) The flowchart of Cell Counting Kit-8 (CCK-8). (B) The EdU-DNA synthesis assays were used to evaluate cell proliferation in GBM cells after ISP-I treatment for 24 h in a dose-dependent manner. Scale bars = 50 µm. (C-D) The apoptosis of GBM cells after treatment with varying doses of ISP-I was determined by Western blotting (C) and FACS-based Annexin-V/PI double staining (D). (E-F) The effect of ISP-I on cell DNA damage was measured by TUNEL assay (E, scale bars = 50 µm) and γ-H2AX immunofluorescence staining (F, scale bars = 20 µm). (G) CompuSyn software was adopted to generate the curves. The Fa-*CI* plots showed the value of combination index (*CI*) in every fractional effect, and ISP-I was synergized with TMZ (*CI* < 1). Fa: inhibition rate. Data: mean ± SD; Significance was calculated with one-way analysis of variance (ANOVA) test. *P < 0.05; **P < 0.01; ***P < 0.001; ns, not significant.


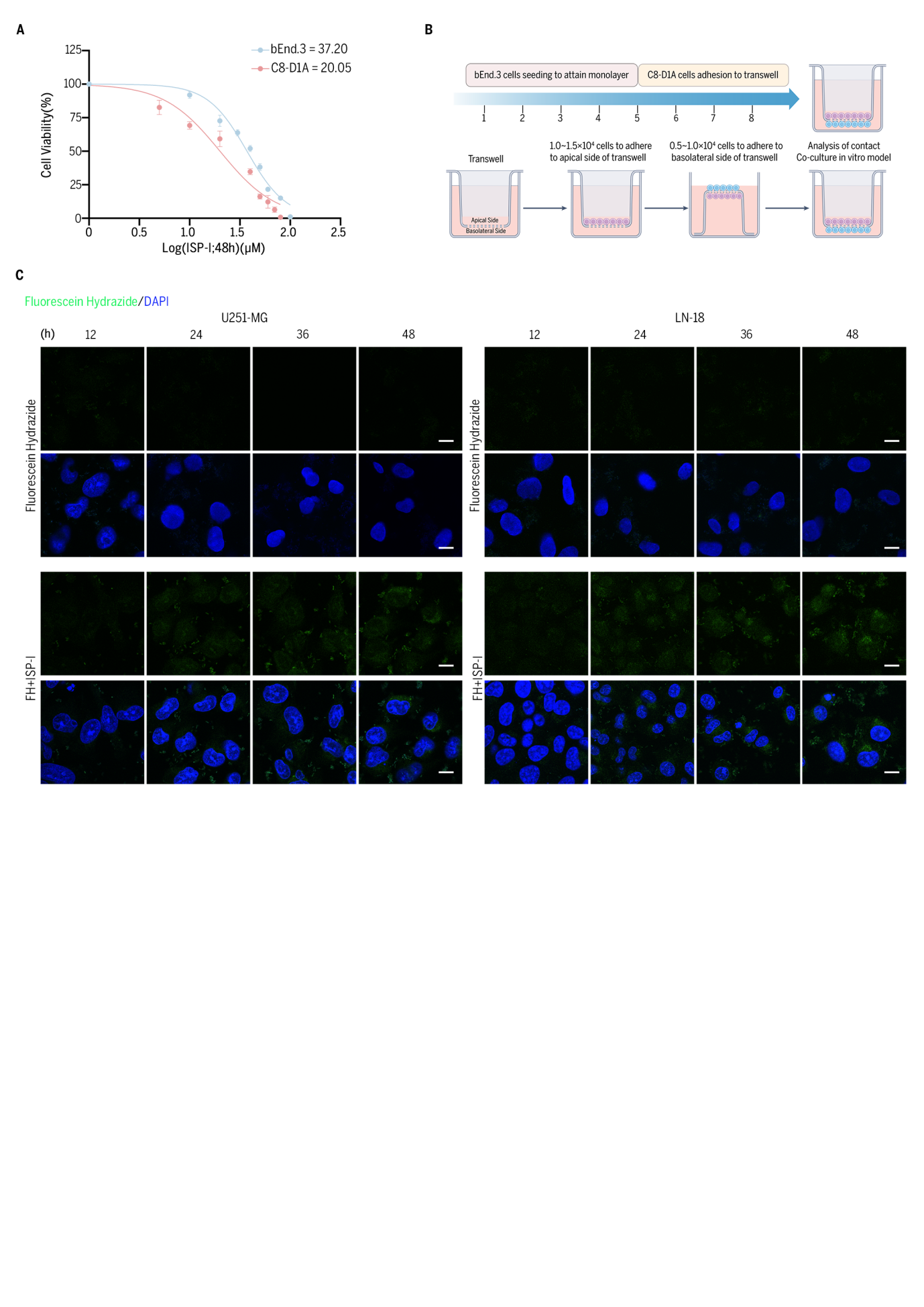


**Fig. S2.** A conventional BBB model was constructed to evaluate the ability of ISP-I to penetrate the BBB *in vitro*. (A) ISP-I was used to treat the bEnd.3 cells and C8-D1A cells for 48 h, and the IC50 was shown. ISP-I (6 µM) had a minimal cytotoxic effect on bEnd.3 cells and C8-D1A cells (n = 3). (B) Schematic timeline representation of bEnd.3 and C8-D1A cell seeding on apical and basolateral sides of the Transwell to attain a contact co-culture in vitro BBB model. (C) Fluorescence imaging of GBM cells in the lower chamber after incubating ISP-I (6.0 µM) in the upper chamber for various times. The transport of ISP-I across the BBB was evaluated by incubation with fluorescein hydrazide (FH, 20 µM) for 1 h at 37 ℃. Scale bars = 20 µm. Data: mean ± SD.


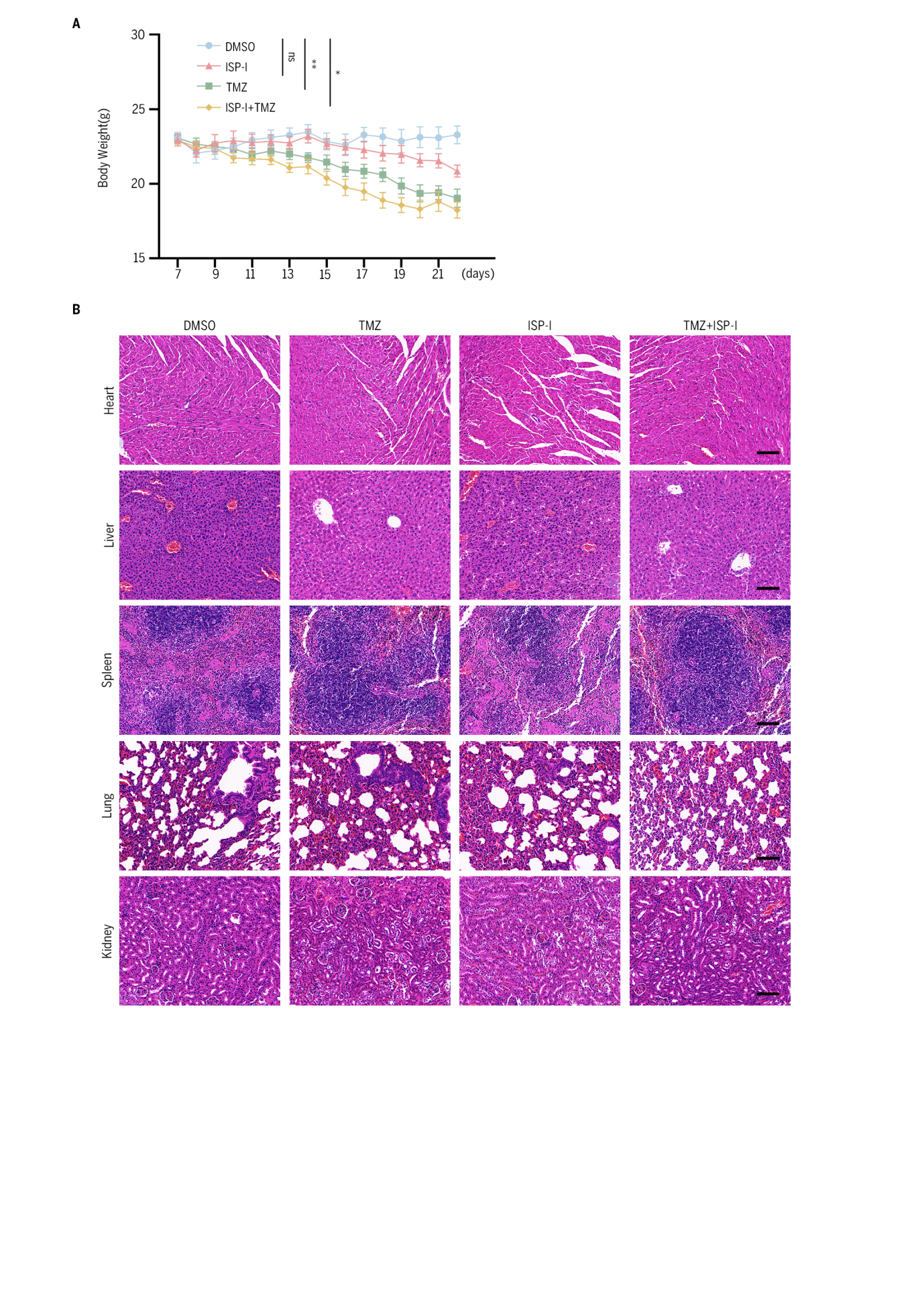


**Fig. S3.** (A) The tumor-bearing mice were divided into four groups, including DMSO group, TMZ group, ISP-I group and TMZ + ISP-I group. Each group of mice was intervened according to the group, and the body weight of mice was monitored daily (n = 8). (B) Representative images of HE staining of major organs from all groups of mice. Scale bars = 200 µm. Data: mean ± SD; Significance was calculated with one-way ANOVA test. *p < 0.05; **p < 0.01; ns, not significant..

**
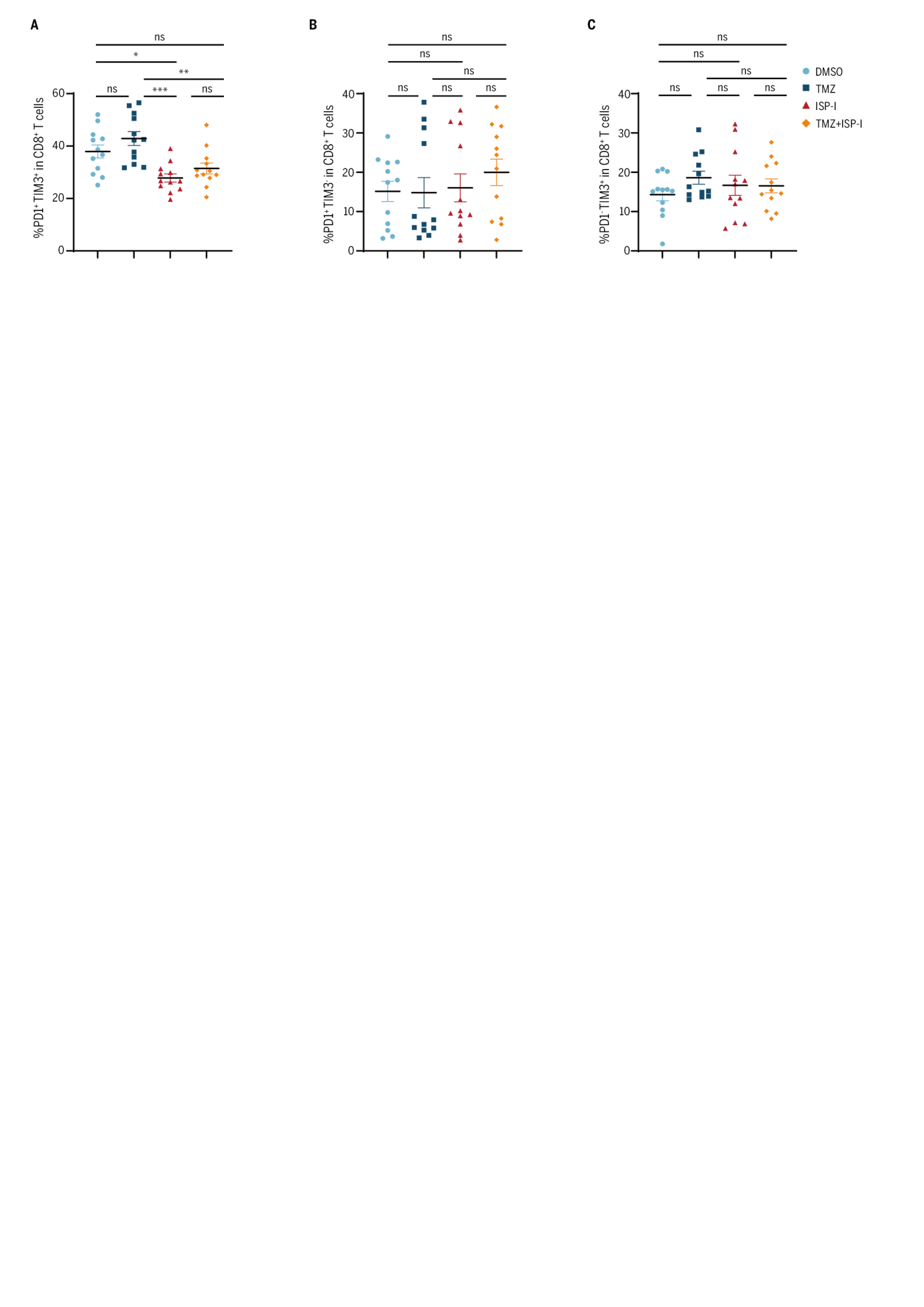
**

**Fig. S4.** ISP-I activates antitumor immune responses in mice. The different exhausted states of tumor infiltrating CD8^+^ T cells in GBM tissues isolated from tumor-bearing mice in each group. Statistical chart of the expression of terminally differentiated exhausted CD8^+^ T cells (A, PD1^+^TIM3^+^), PD1^+^TIM3^−^ CD8^+^ T cells (B), and PD1^−^TIM3^+^ CD8^+^ T cells (C) in tumor infiltration each group of mice (n = 12) were showed. Data: mean ± SD; Significance was calculated with one-way ANOVA test. *P < 0.05; **P < 0.01; ***P < 0.001; ns, not significant.


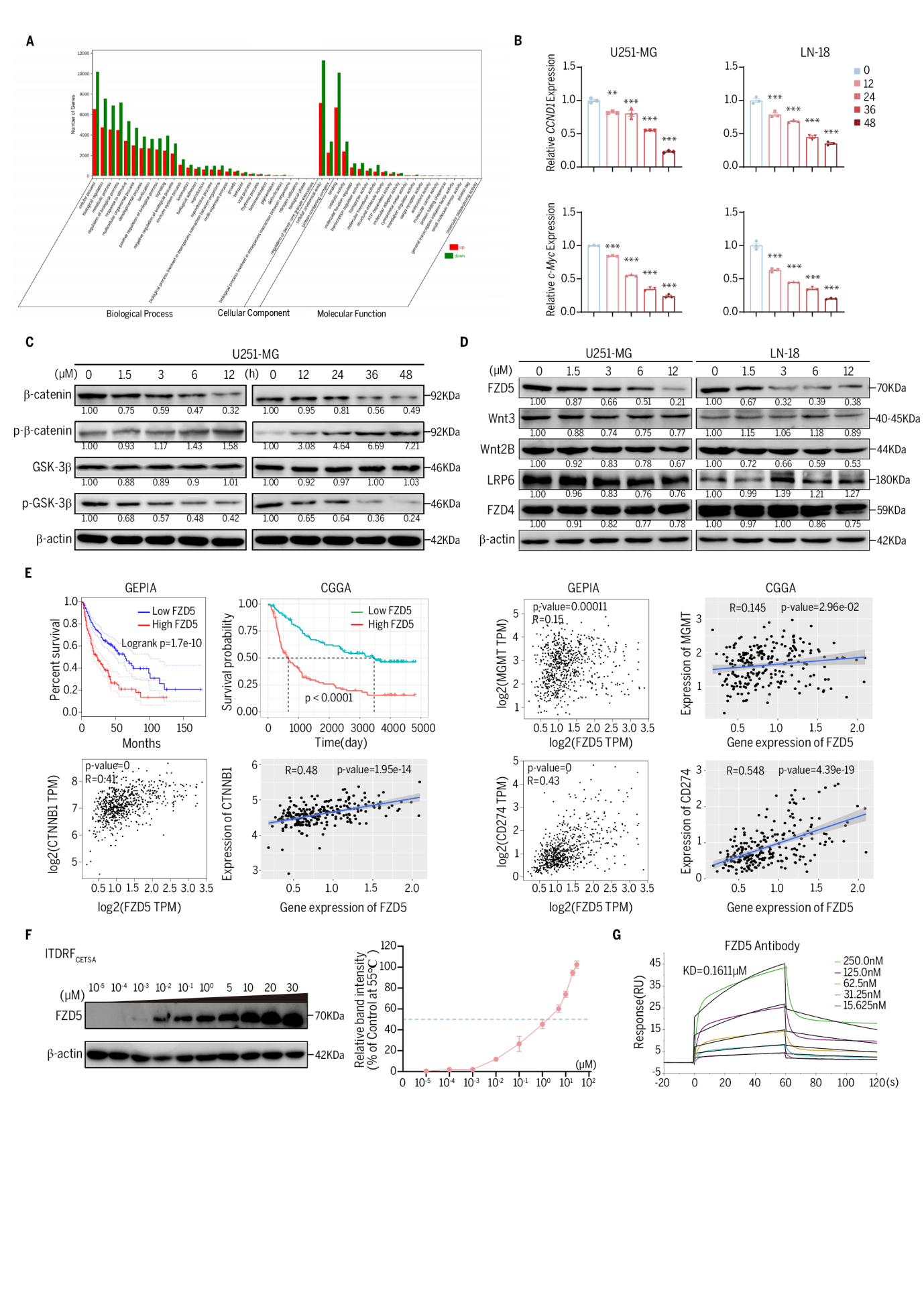


**Fig. S5.** ISP-I targets FZD5 to inhibit the Wnt/β-catenin signaling pathway. (A) Bar chart of Gene Ontology (GO) terms classified by biological process (BP), cellular component (CC), and molecular function (MF). The y-axis was the number of differentially expressed genes. (B) The mRNA levels of the target gene *CCND1* and *Myc* of the Wnt/β-catenin signaling pathway were measured through qRT-PCR after treatment at different time points (n = 3). (C) The ISP-I role in Wnt/β-catenin signaling pathway key protein level was measured through western blotting following ISP-I treatment at different contents in U251-MG cells. (D) The protein level of positive protein based on molecular docking screening was measured through Western Blotting. (E) Survival analysis and gene correlation analysis. The online databases, including Gene Expression Profiling Interactive Analysis (GEPIA) and Chinese Glioma Genome Atlas (TCGA), were used to analyze the disease-free survival analysis of FZD5 gene expression (upper left) and the correlation with β-catenin, CD274 and MGMT in glioma. Gene expression correlation analysis was performed between *FZD5* with *CTNNB1* (bottom left), *CD274* (bottom right) and *MGMT* (upper right). The Pearson's correlation analysis was used to determine the correlation coefficient. FZD5 was used for the x-axis, and other genes of interest are represented on the y-axis. (F) ISP-I increased the FZD5 thermal stability in LN-18 cells by concentration-dependent CETSA (n = 3). (G) SPR experiments was conducted to analyze the interaction between FZD5 antibody and FZD5 protein as a positive control. Data: mean ± SD; Significance was calculated with one-way ANOVA test. *P < 0.05; **P < 0.01; ***P < 0.001; ns, not significant.


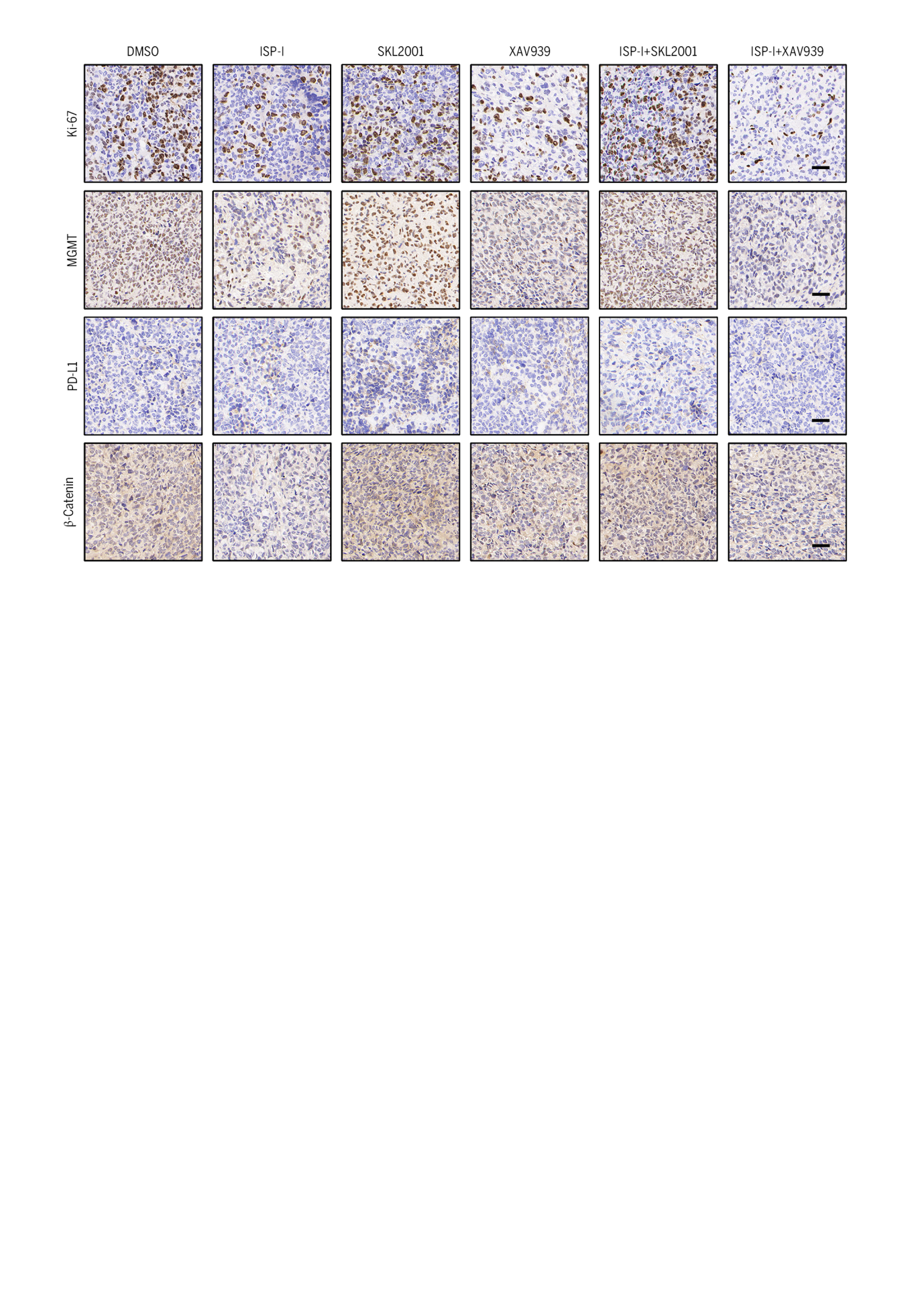


**Fig. S6.** ISP-I inhibits β-catenin to regulate PD-L1 and MGMT expression in subcutaneous tumor-bearing nude mice. Immunohistochemical (IHC) staining of Ki-67, MGMT, PD-L1 and β-catenin in tumor tissues. Scale bars = 20 µm.

**Table S1:** The list of antibodies used in Western blotting.

| **Antibody** | **Company** | **Product code** | **Source** | **Dilution** |
| --- | --- | --- | --- | --- |
| β-actin | Proteintech | 66009-1-Ig | mouse | 1:20000 |
| Cleaved Caspase-3 | Cell Signaling Technology | 9661 | rabbit | 1:1000 |
| Cleaved Caspase-8 | Wanleibio | WL0153 | rabbit | 1:500 |
| Bax | Cell Signaling Technology | 5023 | rabbit | 1:1000 |
| Bcl-2 | Cell Signaling Technology | 2872 | rabbit | 1:1000 |
| γ-H2AX | Abcam | ab2893 | rabbit | 1:2000 |
| PD-L1 | Proteintech | 66248-1-Ig | mouse | 1:5000 |
| MGMT | Abcam | ab39253 | mouse | 1:2000 |
| Calreticulin | Proteintech | 27298-1-AP | rabbit | 1:5000 |
| GADPH | Proteintech | 60004-1-Ig | mouse | 1:50000 |
| ATP1A1 | Proteintech | 14418-1-AP | rabbit | 1:10000 |
| c-Myc | Cell Signaling Technology | 5605 | rabbit | 1:1000 |
| TCF1 | Cell Signaling Technology | 2203 | rabbit | 1:1000 |
| β-Catenin | Cell Signaling Technology | 8480 | rabbit | 1:1000 |
| p-β-Catenin (Ser33/37/Thr41) | Cell Signaling Technology | 9561 | rabbit | 1:1000 |
| GSK-3β | Cell Signaling Technology | 12456 | rabbit | 1:1000 |
| p-GSK-3β (Ser9) | Cell Signaling Technology | 5558 | rabbit | 1:1000 |
| Histone H3 | Proteintech | 68345-1-Ig | mouse | 1:20000 |
| Frizzled 5 | Proteintech | 21519-1-AP | rabbit | 1:1000 |
| Wnt3 | Proteintech | 28156-1-AP | rabbit | 1:500 |
| Wnt2B | Santa Cruz Biotechnology | sc-166502 | mouse | 1:500 |
| LRP6 | Cell Signaling Technology | 3395 | rabbit | 1:1000 |
| FZD4 | Bioworld Technology | BS79010 | rabbit | 1:500 |
| HRP-conjugated Goat anti-Mouse | Proteintech | SA00001-1 | goat | 1:5000 |
| HRP-conjugated Goat anti-Rabbit | Proteintech | SA00001-2 | goat | 1:5000 |

**Table S2:** Sequences of qRT-PCR primers.

| **Item** | **Sequence** |
| --- | --- |
| GADPH | Forward CCAGGTGGTCTCCTCTGACTTC |
|  | Reverse GTGGTCGTTGAGGGCAATG |
| CD274 | Forward GGTGCCGACTACAAGCGAAT |
|  | Reverse TAGCCCTCAGCCTGACATGTC |
| MGMT | Forward ATGAAACGCACCACACTGGA |
|  | Forward AATAGAGCAAGGGCAGCGTT |
| Myc | Forward AGCTTGTACCTGCAGGATCTG |
|  | Reverse GCTCCAAGACGTTGTGTGTTC |
| CCND1 | Forward CTGTGCTGCGAAGTGGAAAC |
|  | Reverse TGTTTGTTCTCCTCCGCCTC |
| β-catenin | Forward TTCTGGTGCCACTACCACAGC |
|  | Reverse TGCATGCCCTCATCTAATGTC |
| GSK-3β | Forward TGGTCGCCATCAAGAAAGTATTG |
|  | Reverse GCGTCTGTTTGGCTCGACTAT |

**Table S3:** The list of antibodies used in immunofluorescence.

| **Antibody** | **Company** | **Product code** | **Source** | **Dilution** |
| --- | --- | --- | --- | --- |
| γ-H2AX | Abcam | ab2893 | rabbit | 1:10000 |
| PD-L1 | Proteintech | 66248-1-Ig | mouse | 1:5000 |
| PD-L1 | Thermo Fisher | PA518337 | goat | 1:200 |
| MGMT | Proteintech | 67476-1-Ig | mouse | 1:300 |
| Calreticulin | Proteintech | 27298-1-AP | rabbit | 1:300 |
| Frizzled 5 | Proteintech | 21519-1-AP | rabbit | 1:300 |
| β-Catenin | Cell Signaling Technology | 8480 | rabbit | 1:100 |
| Alexa Fluor 488 Conjugated anti-Mouse | Cell Signaling Technology | 4408 | goat | 1:1000 |
| Alexa Fluor 488 Conjugated anti-Rabbit | Cell Signaling Technology | 4412 | goat | 1:1000 |
| Alexa Fluor 647 Conjugated anti-Mouse | Cell Signaling Technology | 4410 | goat | 1:1000 |
| Alexa Fluor 555 Conjugated anti-Goat | Abcam | ab150130 | donkey | 1:1000 |
| DyLight 554 Phalloidin | Cell Signaling Technology | 13054 | − | 1:200 |
| DAPI | Cell Signaling Technology | 4083 | − | 1:10000 |

−not applicable.

**Table S4:** The list of antibodies used in flow cytometry.

| **Antibody** | **Company** | **Product code** | **Source** | **Dilution** |
| --- | --- | --- | --- | --- |
| Calreticulin | Proteintech | 27298-1-AP | rabbit | 1:300 |
| Alexa Fluor 488 Conjugated anti-Rabbit | Cell Signaling Technology | #4412 | goat | 1:1000 |
| Rabbit IgG control Polyclonal antibody | Proteintech | 30000-0-AP | rabbit | 1:5000 |
| Fixable Viability Stain 780 | BD Pharmingen | 565388 | rat | 1:1000 |
| FITC Rat Anti-Mouse CD45 (30-F11) | BD Pharmingen | 553079 | rat | 0.2ug/test |
| BV510 Hamster Anti-Mouse CD3e (145-2C11) | BD Pharmingen | 563024 | hamster | 0.2ug/test |
| APC-Cy7 Rat Anti-Mouse CD8a (53-6.7) | BD Pharmingen | 557654 | rat | 0.6ug/test |
| BV421 Hamster Anti-Mouse CD279 (PD-1)(J43) | BD Pharmingen | 562584 | hamster | 0.6ug/test |
| APC anti-mouse CD366 (Tim-3) | BioLegend | 119706 | rat | 0.5ug/test |

**Table S5:** Sequences of ChIP primers.

| **Antibody** | **Target gene** | **Site** | **Sequence** |
| --- | --- | --- | --- |
| LEF1 | CD274 | 1 | F: TGGGAAAATGAATGGCTGAA |
|  |  |  | R: AGTTGCTGATGGGAATTGAGG |
|  |  | 2 | F: CCCTGGGTCTTGACCATTTT |
|  |  |  | R: GACACACACATGTACAACAAGTTTCA |
|  |  | 3 | F: AAGGTAAAATCAAGGTGCGTTCA |
|  |  |  | R: TTTCACCGGGAAGAGTTTCG |
|  | MGMT | 1 | F: TAGTGTGGTGTGGTGCTGGT |
|  |  |  | R: TATACTCATCGCCTTGGAACTG |
|  |  | 2 | F: GGGCCTAGTTTATTTCCCACA |
|  |  |  | R: AGGATTTGCTTTGGGATGAG |
|  |  | 3 | F: GACCGGGATTCTCACTAAGC |
|  |  |  | R: GGCTCTGTGCCTTAGTTTGC |
| TCF7 | CD274 | 1 | F: GCAAATTCCGTTTGCCTCAT |
|  |  |  | R: CCTCCTAGATGGCCTGGATG |
|  |  | 2 | F: TCGAGGAACTTTGAGGAAGTCA |
|  |  |  | R: GACCCATATGGCTTTGGTTTTT |
|  |  | 3 | F: AAGGTAAAATCAAGGTGCGTTCA |
|  |  |  | R: TTTCACCGGGAAGAGTTTCG |
|  | MGMT | 1 | F: CAGTCGGCACTCTGATTCCA |
|  |  |  | R: TGCTATACACCTGGCCACTTACA |
|  |  | 2 | F: CCAGAAGTTTGAAACCCAGGTG |
|  |  |  | R: GCCTAAGAGGACAGACCCTTCC |
|  |  | 3 | F: GACCGGGATTCTCACTAAGC |
|  |  |  | R: GGCTCTGTGCCTTAGTTTGC |

F = Forward; R = Reverse.

**Table S6:** Molecular docking results between ISP-I and the potential target protein of Wnt/β-catenin signaling pathway.

| **Protein** | **PDB or AlphaFold DB ID.** | **Hydrogen bond** | **Vina score (kcal/mol)** |
| --- | --- | --- | --- |
| FZD5 | 5URY | ASN22､ARG58､GLY18､TYR125 | -6.06 |
| Wnt3 | AF-P56703 | − | -5.62 |
| Wnt2B | AF-Q93097 | LYS108 | -5.04 |
| LRP6 | 3S94 | ASP746 | -4.78 |
| FZD4 | 5BPB | − | -4.65 |
| Wnt8A | 7KC4 | − | -3.74 |
| Wnt7B | AF-P56706 | − | -3.5 |
| LRP5 | AF-O75197 | − | -3.27 |
| FZD2 | 6C0B | GLN148 | -2.38 |
| FZD6 | 8JHB | THR204 | -2.34 |
| Wnt4 | AF-P56705 | − | -2.24 |
| FZD7 | 7EVQ | HIS519 | -2.2 |
| Wnt3A | 7DRT | − | -2.17 |
| FZD3 | 8JHI | − | -2.04 |
| Wnt16 | AF-Q9UBV4 | − | -1.88 |
| Wnt5B | AF-Q9H1J7 | − | -1.71 |
| Wnt1 | AF-A0A087WXR9 | − | -1.32 |
| FZD1 | 8J8N | − | -1.00 |
| Wnt7A | AF-O00755 | − | -0.91 |
| Wnt5A | AF-P41221 | − | -0.91 |
| Wnt8B | AF-Q93098 | − | -0.74 |
| Wnt2 | AF-P09544 | − | -0.67 |
| FZD9 | AF-O00144 | UNL1 | -0.64 |
| Wnt9B | AF-E7EPC3 | − | -0.63 |
| Wnt11 | AF-O96014 | GLY50 | -0.48 |
| FZD8 | 8X0T | ARG235 | -0.29 |
| FZD10 | AF-Q9ULW2 | ARG443 | -0.24 |
| Wnt6 | AF-Q9Y6F9 | − | -0.04 |
| Wnt10A | AF-Q9GZT5 | − | 0.24 |
| Wnt9A | AF-O14904 | − | 0.64 |
| Wnt10B | AF-O00744 | − | 1.02 |

−not applicable.

**Table S7:** JASPAR score on predictive site of the promoter-binding region of the target gene.

| **TFs** | **Sequence ID** | **Site** | **Start** | **End** | **Predicted Sequence** | **Score** |
| --- | --- | --- | --- | --- | --- | --- |
| **LEF1** | **CD274** | 1 | 178 | 192 | AAAGATGAACAAAAA | 4.8211255 |
|  |  | 2 | 622 | 636 | GTAGATCTAAGTATT | 9.107637 |
|  |  | 3 | 1811 | 1825 | TTTGCTTTAATCTTC | 12.382987 |
|  | **MGMT** | 1 | 310 | 324 | TAATATGGAAGGATA | 2.9448733 |
|  |  | 2 | 658 | 672 | GAACCTCAGAGGATC | 4.3799734 |
|  |  | 3 | 1773 | 1787 | TGACAGGAAAAGGTA | 1.6415718 |
| **TCF1** | **CD274** | 1 | 328 | 334 | CTTTCAT | 7.242653 |
|  |  | 2 | 1488 | 1494 | CTTTGAG | 9.976732 |
|  |  | 3 | 1815 | 1821 | CTTTAAT | 7.185436 |
|  | **MGMT** | 1 | 149 | 155 | CTCTGAT | 7.074047 |
|  |  | 2 | 1113 | 1119 | GTTTGAA | 5.8800645 |
|  |  | 3 | 1716 | 1722 | CTTTCAG | 4.4729767 |
